# Anverenes B–E, New Polyhalogenated Monoterpenes from the Antarctic Red Alga *Plocamium cartilagineum*

**DOI:** 10.3390/md17040230

**Published:** 2019-04-17

**Authors:** Andrew J. Shilling, Jacqueline L. von Salm, Anthony R. Sanchez, Younghoon Kee, Charles D. Amsler, James B. McClintock, Bill J. Baker

**Affiliations:** 1 Department of Chemistry, University of South Florida, 4202 E. Fowler Ave., CHE205, Tampa, FL 33620, USA; ashillin@mail.usf.edu (A.J.S.); j.vs.fries@gmail.com (J.L.v.S.); 2 Department of Cell Biology, Microbiology, and Molecular Biology, University of South Florida, 4202 E. Fowler Ave., ISA2015, Tampa, FL 33620, USA; ars1@mail.usf.edu (A.R.S.); ykee@usf.edu (Y.K.); 3 Department of Biology, University of Alabama at Birmingham, Birmingham, AL 35294, USA; amsler@uab.edu (C.D.A.); mcclinto@uab.edu (J.B.M.)

**Keywords:** halogenated monoterpenes, anverenes, *Plocamium cartilagineum*

## Abstract

The subtidal red alga *Plocamium cartilagineum* was collected from the Western Antarctic Peninsula during the 2011 and 2017 austral summers. Bulk collections from specific sites corresponded to chemogroups identified by Young et al. in 2013. One of the chemogroups yielded several known acyclic halogenated monoterpenes (**2**–**5**) as well as undescribed compounds of the same class, anverenes B–D (**6**–**8**). Examination of another chemogroup yielded an undescribed cyclic halogenated monoterpene anverene E (**9**) as its major secondary metabolite. Elucidation of structures was achieved through one-dimensional (1D) and 2D nuclear magnetic resonance (NMR) spectroscopy and negative chemical ionization mass spectrometry. Compounds **1**–**9** show moderate cytotoxicity against cervical cancer (HeLa) cells.

## 1. Introduction

**Scheme 1:**
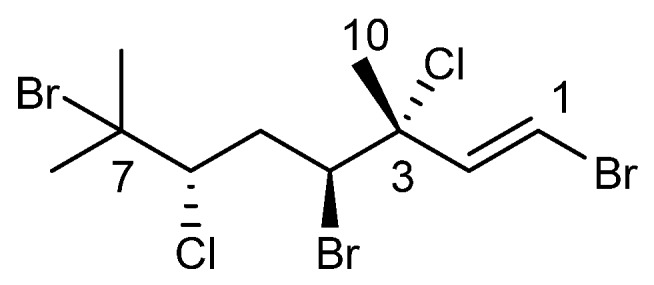
The chemical structure of anverene A (**1**).

*Plocamium cartilagineum* is a broadly distributed red alga that contributes to the structure of algal-dominated coastal benthic ecosystems of the Western Antarctic Peninsula [1]. Although there is no currently accepted alternate species name, *P. cartilagineum* in Antarctica is known to be a distinct species from what is called *P. cartilagineum* in other parts of the world [2]. This rhodophyte is known to produce many different polyhalogenated monoterpenes, including anverene (**1**, Scheme 1), which has been shown to convey ecological relevance as a feeding deterrent toward sympatric macroalgal consumers [3,4]. Previous studies have shown that chemical diversity among phenotypes of this alga is high, with specific chemotypes (chemogroups [1]) displaying different halogenated terpenes with variable relative abundances. Patterns of chemogroup variability have been linked through metabolomics with site-specificity, presumably driven by ecological interactions [1,3,5]. In 2013, Young et al. showed that within a collection of 21 individual algal specimens, there existed two distinct genotypes and five distinct chemotypes [1]. The current study was carried out to identify and describe the major compounds present in two of the chemogroups previously identified, as well as report new structures found during the analysis. 

**Scheme 2:**
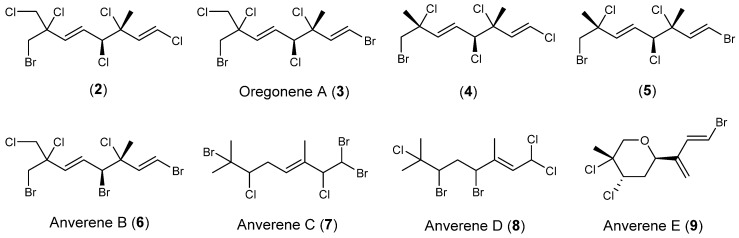
Chemical structures of halogenated monoterpenes isolated from Antarctic *Plocamium cartilagineum*.

The study began with a bulk collection of *Plocamium cartilagineum* from a 2011 Antarctic field season obtained at Gamage Point on Anvers Island. Upon extraction and gas chromatography/mass spectrometry (GC/MS) analysis, this bulk collection was shown to share many metabolites with previously reported chemogroup 4 [1]. This chemically rich chemogroup produces primarily linear polyhalogenated monoterpenes and yielded known compounds **2**–**5** (Scheme 2), and previously unreported anverene B (**6**) as its most abundant metabolites, as well as previously unreported anverenes C and D (**7** and **8**) as minor metabolites [6,7,8].

Another field collection was undertaken in the austral summer of 2017 from Norsel Point, which was shown to match the metabolomic profile of the previously reported chemogroup 1, the chemogroup with the least amount of similarity to chemogroup 4 of any of the previously identified chemotypes and one that also belonged to a different genotype [1]. Upon extraction and fractionation, chemogroup 1 was shown to contain small amounts of **2**, however its most abundant metabolite was the previously unreported cyclic monoterpene anverene E (**9**), which features a terminal diene not previously reported from this taxon. Fractionation of each algal specimen was guided by GC/MS and nuclear magnetic resonance (NMR) spectroscopy resulting in purified compounds that are traceable to the crude extracts, leading us to the conclusion that no compounds are artifacts of the isolation. Herein we report the characterization of the three new compounds from chemogroup 1, anverenes B–D (**6**–**8**) and one from chemogroup 4, anverene E (**9**) as well as HeLa cytotoxicity for compounds **1**–**9**. 

## 2. Results and Discussion

**Figure 1:**
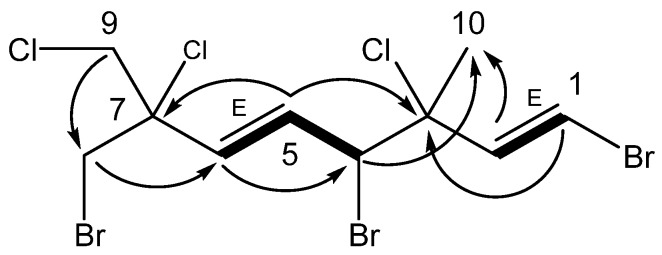
Key HMBC (→) and COSY (▬) correlations establishing the planar structure for anverene B (**6**), double bond geometry based on ^3^*J*_HH_.

Anverene B (**6**) was obtained as a clear oil with spectral data similar to that of **2** and **3**. A molecular formula of C_10_H_12_Br_3_Cl_3_ was established from high resolution negative chemical ionization mass spectrometry (HRNCIMS) ([M − H]^−^: m/z 472.7477, calc. 472.7482), corroborated by ^1^H and ^13^C NMR spectra (Table 1). Key ^1^H NMR signals (Figure 1) include a vinyl methine doublet, H-1 (δ_H_ 6.56), coupled in the COSY spectrum to a second vinyl methine doublet, H-2 (δ_H_ 6.41), the latter of which could be linked through HMBC correlations to a doublet secondary alkyl methine, H-4 (δ_H_ 4.60). Additional vinyl methine signals H-5 (δ_H_ 6.22) and H-6 (δ_H_ 6.00) were established through COSY correlations as part of an isolated spin system bounded by C-4 and C-6. Two isolated doublet methylene groups, H_2_-8 (δ_H_ 3.83, 3.87) and H_2_-9 (δ_H_ 3.91, 3.98), correlated to C-6 and C-7, as well as to each other, completing the western portion of a monoterpene. The last unaccounted ^1^H NMR signal, a singlet methyl, H_3_-10 (δ_H_ 1.81), was found through HMBC correlations to C-2 through C-4, as occupying quaternary C-3, yielding the planar structure of anverene B (Figure 1). 

Halogen regiochemistry was primarily defined using ^13^C NMR shifts. The halogen bearing carbons in the eastern portion of anverene B (**6**) including C-1 (δc 110.3), C-3 (δc 70.9), and C-4 (δc 59.3) were matched closely to the carbon shifts reported in anverene A (**1**) at the same positions, and were assigned the same substituents; alternate halogenation patterns are not supported by comparison of their carbon shifts to previously published *P. cartilagineum* compounds nor calculated chemical shifts (Table S1) [4,6]. Similarly, the halogen bearing carbons in the western portion of anverene B including C-7 (δc 68.8), C-8 (δc 37.2), and C-9 (δc 49.6) matched closely to the carbon shifts reported in **2** at the same positions [6].

The stereochemical configuration of anverene B (**6**) was studied using ^3^*J*_HH_ and comparison with previous *Plocamium cartilagineum* data sets [4,8]. The alkenes were both determined as *E* based on ^3^*J*_HH_ = 13.6 and 15.3 (Table 1) [8,9]. The relative configuration at C-3 and C-4 was determined by comparison of the carbon shift of C-10 (δc 25.9) with anverene (**1**) (C-10 δc 25.5) suggesting the same (R*,S*) configuration [4]. The C-10 methyl group was first noted by Crews as an indicator of differentiation between the (*R*,*S*) and (*R*,*R*) configurations at positions C-3 and C-4 within compounds of the same scaffold. He reported three (*R*,*S*) variants with carbon shifts ranging from δc 24.6–25.4 ppm and two (*R*,*R*) variants with carbon shifts ranging from 28.0–28.4 ppm, specifically making note of the 3 ppm downfield shift difference between a pair of (*R,S*) and (*R,R*) diastereomers at positions C-3 and C-4 [7]. The values seen in anverenes A and B are in agreement with those assignments. The configuration of C-7 was recalcitrant to spectroscopic methods.

**Scheme 3:**
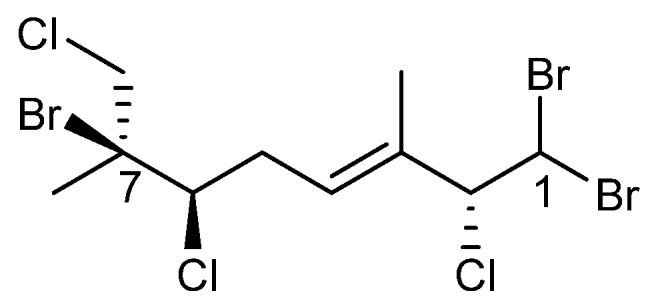
The chemical structure of (3*E*,2*R*,6*R*,7*S*)-1,1,7-tribromo-2,6,8-trichloro-3,7-dimethyloct-3-ene (**10**).

**Figure 2:**
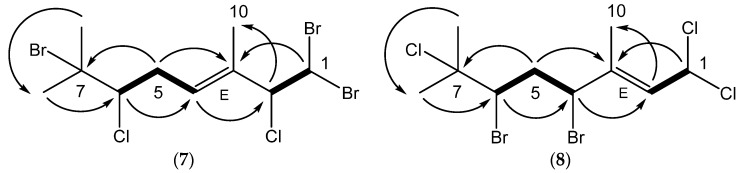
Key HMBC (→) and COSY (▬) correlations establishing the planar structures for anverenes C (**7**) and D (**8**).

Anverene C (**7**) was obtained as a clear oil with spectral data similar to that of (3*E*,2*R*,6*R*,7*S*)-1,1,7-tribromo-2,6,8-trichloro-3,7-dimethyloct-3-ene (**10**) (Scheme 3) [10]. A formula of C_10_H_15_Br_3_Cl_2_ was established from HRNCIMS ([M − H]^−^: m/z 440.8029, calc. 440.8028). Anverene C departed from the anverene A (**1**) and B (**6**) motif of the terminal vinyl bromide, displaying a doublet methine, H-1 (δ_H_ 5.68), on an alkyl-like carbon (δc 45.1) coupled in the COSY spectrum to a similar aliphatic doublet, H-2 (δ_H_ 4.80) with a carbon shift indicative of an electronegative substituent (δc 73.4). This spin system was extended through HMBC correlation of H-2 to quaternary vinyl carbon C-3 (δc 134.0) and methine C-4 (δc 130.0), the latter bearing a triplet vinyl proton H-4 (δ_H_ 5.83). A pair of diastereotopic alkyl protons H-5_a_ (δ_H_ 2.53) and H-5_b_ (δ_H_ 3.15) and methine H-6 (δ_H_ 4.03) were established through COSY correlations as part of a spin system including C-4 through C-6. H_2_-5 could be assigned contiguous to C-4 by HMBC correlations of H-5_a_ and H-5_b_ to C-3 and the reciprocal H-4 to C-6. Singlet methyl H_3_-10 (δ_H_ 1.76) displayed HMBC correlations to C-2 through C-4, establishing the eastern portion of anverene C. The remaining two singlet methyl groups, H_3_-8 (δ_H_ 1.80) and H_3_-9 (δ_H_ 1.93) were found on a quaternary alkyl carbon-bearing-heteroatom, C-7 (δ_C_ 70.5) based on HMBC correlations of their respective protons to C-7, and integrated into the remainder of the monoterpene by virtue of an HMBC correlation to C-6, resulting in the planar structure for anverene C as depicted in Figure 2. Configuration of the ∆^2^ alkene of anverene C was determined as *E* based on observation of NOE enhancement of H-2 upon irradiation of H-4.

Halogen regiochemistry was defined, as with anverene B (**6**), based on ^13^C NMR data as well as ^1^H NMR shifts. The C-1 methine (δc 45.1) of anverene C (**7**) requires two halogens; the shift is reminiscent of that for C-1 of **10** (δc 44.2) and incompatible with the value expected for a gem-dichloro (>60 ppm; see anverene D (**8**), below). C-2 (δc 73.4) of anverene C is downfield compared to C-2 (δc 64.9) in **10**, however a similar proton shift of methine H-2 in **7** and **10** (δ_H_ 4.80 and 4.73, respectively) argues for the presence of chlorine at C-2 in both [10]. The two remaining halogens, one chlorine atom and one bromine atom, must fill the remaining open valences at C-6 and C-7. The carbon shifts found in anverene C (C-6, δc 68.8; C-7, δc 70.5) match those found in anverene A (C-6, δc 69.2; C-7, δc 66.3). The proton shifts of the flanking geminal dimethyl group similarly match (anverene C: H_3_-8 (δ_H_ 1.80) and H_3_-9 (δ_H_ 1.93); anverene A: H_3_-8 (δ_H_ 1.81) and H_3_-9 (δ_H_ 1.92)) [4]. Compare the proton shifts of the gem-dimethyl group of anverene D, where the chloride and bromide have flipped positions, resulting in a 0.1 ppm upfield shift (Table 2). The rotatable nature of the acyclic scaffold rendered the stereochemical determination at C-2 and C-6 unattainable using spectroscopic methods. 

**Scheme 4:**
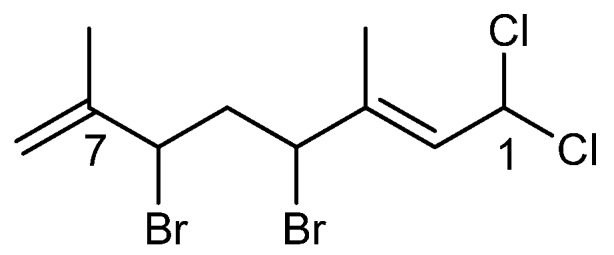
The chemical structure of plocoralide B (**11**).

Anverene D (**8**) was obtained as a clear oil with spectral data similar to that of anverene C (**7**) and certain shifts bearing close resemblance to plocoralide B (**11**) (Scheme 4) [11]. A formula of C_10_H_15_Br_2_Cl_3_ was established from HRNCIMS ([M − H]^−^: m/z 396.8535, calc. 396.8533). Anverene D displays a doublet methine, H-1 (δ_H_ 6.40), on an sp^3^ carbon with a shift (δc 66.6) indicative of substitution by electronegative groups but differing from anverene C in that it is coupled in the COSY spectrum to a doublet vinyl proton H-2 (δ_H_ 6.03) situated on an sp^2^ methine carbon (C-2, δc 128.0). This spin system was extended through HMBC correlation of H-1 to quaternary olefinic carbon C-3 (δc 138.2), and further HMBC correlation of H-2 to a methine C-4 (δc 57.8), the latter bearing a proton split as a doublet of doublets (H-4, δ_H_ 4.81). A pair of diastereotopic alkyl protons H-5_a_ (δ_H_ 2.13) and H-5_b_ (δ_H_ 2.88) and methine H-6 (δ_H_ 4.43) were established through COSY correlations as part of a spin system including C-4 through C-6. Further expansion of that partial structure was facilitated by HMBC correlation of a singlet methyl group (H_3_-10), (δ_H_ 1.94) to C-2 through C-4. The remaining two singlet methyl groups, H_3_-8 (δ_H_ 1.71) and H_3_-9 (δ_H_ 1.84) displayed HMBC correlation to a quaternary aliphatic carbon, C-7 (δc 70.8) as well as C-6, completing the planar structure for anverene D as depicted in Figure 2. Configuration of the ∆^2^ alkene of anverene D was determined as *E* based on observation of NOE enhancement of H-4 upon irradiation of H-2.

Halogen regiochemistry was defined, as with anverenes B (**6**) and C (**7**), based on ^13^C NMR as well as ^1^H NMR shifts. The halogen bearing carbon C-1 of anverene D (**8**) was much farther downfield in both carbon shift (δc 66.6) and proton shift of the methine H-1 (δ_H_ 6.40) in relation to the analogous carbon shift (δc 45.1) and proton shift of the methine H-1 (δ_H_ 5.68) of anverene C at the same C-1 position, and closely resembled the C-1 (δc 66.5) and H-1 (δ_H_ 6.36) shifts reported in plocoralide B (**11**), leading us to assign a geminal dichloride substituent that accounted for two of the three chlorine atoms in the molecular formula. The halogen bearing carbon C-4 (δc 57.8) of anverene D was similar to the carbon shift reported in anverene A (**1**) (δc 59.8) and plocoralide B (**11**) (δc 55.9) at the same position C-4, and hence could be assigned the same bromine substituent [10]. The halogen bearing carbon C-6 (δc 62.7) was upfield in relation to the carbon shift reported in anverene A at the chlorine bearing C-6 (δc 69.2) position, and was assigned a bromine substituent, accounting for the second of the two bromine atoms in the formula. Finally, the halogen bearing carbon C-7 (δc 70.8) was downfield in relation to the carbon shift reported in anverene A at the bromine bearing C-7 (δc 66.3) position and was assigned a chlorine substituent accounting for the final halogen of the formula. This final assignment is supported by the differences seen in the proton shifts of the flanking geminal dimethyl groups of anverene D (H_3_-8, δ_H_ 1.71; H_3_-10, δ_H_ 1.84), compared to that of anverene A at the same positions (H_3_-8, δ_H_ 1.81; H_3_-9, δ_H_ 1.92) reflecting the greater deshielding effects of the bulkier bromine substituent seen in anverene A in relation to protons occupying the flanking geminal groups [4]. The rotatable nature of the acyclic scaffold rendered the stereochemical determination at C-4 and C-6 impractical using spectroscopic methods.

**Figure 3:**
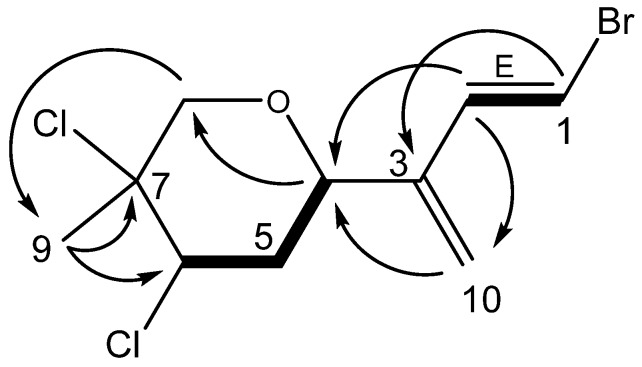
Key HMBC (→) and COSY (▬) correlations establishing the planar structure for anverene E (**9**).

Anverene E (**9**) was obtained as a clear oil with a formula of C_10_H_13_BrCl_2_O established from HRNCIMS ([M + Br]-: m/z 376.8705, calc. 376.8716) and corroborated by ^1^H and ^13^C NMR spectra (Table 3). Key ^1^H NMR signals (Figure 3) include a vinyl methine doublet, H-1 (δ_H_ 6.56), coupled in the COSY spectrum to a second vinyl methine doublet, H-2 (δ_H_ 6.73). This spin system was extended through HMBC correlation of H-1 to quaternary vinyl carbon C-3 (δc 143.2), and further HMBC correlation of H-2 to a methine C-4 (δc 72.0) bearing a proton coupled as a doublet of doublets (H-4, δ_H_ 4.68). A terminal olefin C-10 (δc 116.8) bearing two singlet vinyl protons H-10_a_ (δ_H_ 5.26) and H-10_b_ (δ_H_ 5.28) was established as part of a diene residing in the linear eastern portion of a monoterpene through HMBC correlations of H-2 to C-10 as well as H-10_a_ and H-10_b_ to both C-3 and C-4. A pair of diastereotopic methylene protons H-5_β_ (δ_H_ 2.30) and H-5_α_ (δ_H_ 2.42) and a triplet methine H-6 (δ_H_ 4.52) were established through COSY correlations as part of a spin system including C-4 through C-6. The position of another pair of diastereotopic methylene protons H-8_β_ (δ_H_ 3.65) and H-8_α_ (δ_H_ 4.05) was assigned through HMBC correlations of H_2_-8 to C-4 as well as to C-6, determining its position within a cyclic system comprising the western portion of the monoterpene, with C-4 and C-8 linked through the same electronegative substituent reflected by their similar carbon shifts C-4 (δc 72.0) and C-8 (δc 71.5). Based on the carbon shifts and the oxygen atom in the molecular formula, the cyclic system must be a pyran. The remaining singlet methyl group, H_3_-9 (δ_H_ 1.80) was found on a quaternary alkyl carbon-bearing-heteroatom, C-7 (δc 66.4) based on an HMBC correlation of H_3_-9 to C-7, and integrated into the ring system of the western portion by virtue of HMBC correlations to C-6 and C-8, resulting in the planar structure for anverene E as depicted in Figure 3.

Halogen regiochemistry was defined, as with anverenes B–D (**6**–**8**), based on ^13^C NMR as well as ^1^H NMR shifts. The halogen bearing olefinic carbon of anverene E (**9**) (C-1, δc 108.2) as well as its methine proton (H-1, δ_H_ 6.56) was similar to the carbon (δc 109.7) and proton (δ_H_ 6.58) shifts reported in anverene A (**1**) at the same C-1 position and was assigned analogous bromide substituent accounting for the lone bromine atom in the molecule [4]. The remaining chlorine atoms were assigned to the other two halogenated carbons on the molecule including C-6 which displays a methine proton shift H-6 (δ_H_ 4.52) and adjacent methyl group H_3_-9 (δ_H_ 1.80) similar to the analogous moiety reported in **2** (H-4, δ_H_ 4.48; H_3_-9, δ_H_ 1.75) [6]. 

**Figure 4:**
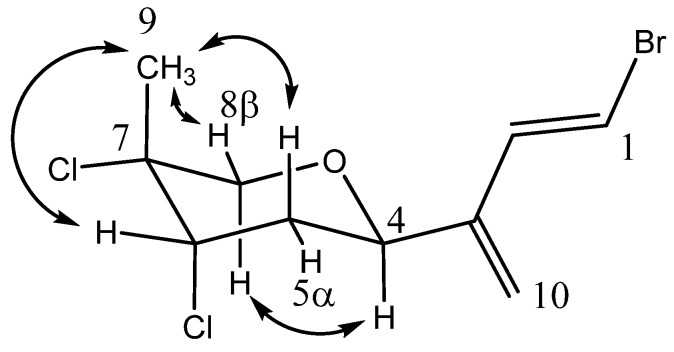
NOESY correlations establishing the relative configuration of anverene E (**9**).

NOESY correlations facilitated the assignment of the relative stereochemical configuration of anverene E (**9**) (Figure 4). A correlation from H-4 (δ_H_ 4.68) to H-8_α_ (δ_H_ 4.05) established the axial orientation of these protons on the α face of the pyran ring and placing the diene chain in the equatorial position. A correlation from H-5_β_ (δ_H_ 2.30) to H_3_-9 (δ_H_ 1.80) then informed the placement of the methyl group as axial and occupying the β face of the pyran ring, an assignment further supported by a correlation to from H_3_-9 to the adjacent equatorial H-8_β_ (δ_H_ 3.65). The equatorial position of H-6 (δ_H_ 4.52) was derived both by its NOESY correlation to the axial H_3_-9 methyl group and through evaluation of the coupling constant (^3^*J*_5β,6_ = 8.4). The alkene at C-1 and C-2 was determined as *E* based on ^3^*J*_1,2_ = 14.1 (Table 3).

Previous investigations into the therapeutic potential of halogenated monoterpenes have yielded a plethora of biological activities ranging from antimicrobial to antitumor properties. The cytotoxic nature of these compounds is likely derived from their original ecological function as feeding deterrents and antifouling agents [3,4,5]. Compound **4** was previously evaluated for its activity against a human esophageal cancer cell line by Knott et al. (2005) who reported an IC_50_ of 9.3 µM. Compounds **1**–**9** were consequently evaluated in vitro against a human cervical cancer cell line (HeLa) and all of the compounds showed low micromolar cytotoxicity, with **2**, **3**, and anverene D (**7**) displaying single-digit micromolar activity (Table 4). Anverene A was on hand from previous investigations [4].

## 3. Conclusions

Subtidal red algae currently lumped in the single species *Plocamium cartilagineum* have historically yielded a large and varied group of halogenated monoterpenes since chemical examination into its metabolome began several decades ago [6]. However, we are now learning that specific trends in this variation can be linked with specific chemical phenotypes produced amongst similar individuals [1]. The results of our investigation indicate that certain chemotypes tend to produce acyclic polyhalogenated monoterpenes while others express primarily cyclic compounds, which may explain some of the variation seen in past inquiries. Our study has also shown that *Plocamium cartilagineum* remains a rich source of new polyhalogenated terpenoid metabolites, warranting further investigation which will undoubtedly yield a greater variety of these unique molecules. Our methodology has shown that examinations limited within specific chemogroups may be a more efficient strategy of targeting specific types of compounds than extraction of haphazardly collected bulk algae and could increase the likelihood of finding novel chemistry. We have demonstrated that these compounds display moderate cytotoxicity towards human cervical cancer cells, justifying further evaluation. 

## 4. Materials and Methods 

### 4.1. General Procedures 

Solvents were obtained from Fisher Scientific Co. (Hampton, NH, USA) and were HPLC grade (>99% purity) unless otherwise stated. MPLC analysis and fractionation were performed on a Teledyne-Isco CombiFlash system equipped with an evaporative light scattering detector (ELSD). HPLC analysis and fractionation was performed on a Shimadzu LC20-AT or LC10-AT system equipped with a Shimadzu ELSD II or Sedex 75 ELSD respectively, using semi-preparative or preparative silica ((250 × 10 mm, 5 μm) or (250 × 21.2 mm, 5 μm)) conditions. GC/MS analysis was performed on an Agilent 7890A GC (Santa Clara, CA, USA) coupled to an Agilent 7200 accurate mass QToF with negative chemical ionization utilizing methane as the reagent gas on a Zebron ZB-5HT Inferno (30 m × 0.25 mm, 0.25 μm film thickness) column. NMR spectra were acquired in CDCl_3_ with residual solvent referenced as an internal standard (δ_H_ 7.27 ppm; δ_C_ 77.0 ppm) for ^1^H and ^13^C NMR spectra, respectively. The ^1^H NMR spectra were recorded on a Varian 500 MHz or 600 MHz direct-drive instrument equipped with cold-probe detection and ^13^C NMR spectra were recorded at 125 MHz. UV absorptions were measured by an Agilent Cary 60 UV-Vis spectrophotometer in CH_3_OH, while IR spectra were recorded with an Agilent Cary 630 FTIR. Optical rotations were measured using an AutoPol IV polarimeter (Hackettstown, NJ, USA) at 589 nm utilizing a 10 mm path length cell. 

### 4.2. Collection of Plocamium cartilagineum

Algal samples were collected using SCUBA from sites around Palmer Station, Antarctica in the austral summers of 2011 and 2017. The collection sites chosen were Gamage Point (64°46.345′ S 64°02.915′ W) and Norsel Point (64°45.674′ S, 64°05.467′ W) at depths between 5–35 m. Samples were frozen and transported back to the University of South Florida at −70 °C and stored at −20 °C until further processing. Herbarium vouchers for the *Plocamium cartilagineum* collections are maintained in the herbarium of CDA at the University of Alabama at Birmingham and available for loan to other herbaria on request.

### 4.3. Extraction and Isolation of Natural Products 

Compounds **2**–**8** were isolated from 2.2 kg of frozen *Plocamium cartilagineum* samples collected from Gamage Point in 2011. The bulk mass was extracted three times with 3:1 dichloromethane:methanol (ACS grade) and the combined extracts subjected to a dichloromethane:water partition and then concentrated in vacuo. The lipophilic extract (56.5 g) was fractionated using MPLC utilizing a hexane to ethyl acetate gradient to yield 14 fractions of increasing polarity. Fraction C (third fraction) (2.9 g) was shown by GC/MS to be rich in characteristic halogenated terpenoids and was subjected to normal phase HPLC conditions utilizing a hexane to 5% 1-chlorobutane gradient over 90 min on two semi-preparative silica columns connected in series. These unique normal phase separation conditions afforded (in retention time order) anverene C (**7**, 1.2 mg), anverene D (**8**, 0.5 mg), compound **4** (18 mg), compound **5** (8 mg), compound **2** (120 mg), oregonene A (**3**, 25 mg), and anverene B (**6**, 12 mg). 

Anverene E (**9**) was isolated from 487 g of frozen *Plocamium cartilagineum* samples collected from Norsel Point in 2017. The bulk mass was extracted three times with 3:1 dichloromethane:methanol (ACS grade) and the combined extracts subjected to a dichloromethane:brine partition and then concentrated in vacuo. The lipophilic extract (13 g) was fractionated using MPLC utilizing a hexane to 20% ethyl acetate gradient to yield 18 fractions of increasing polarity. Fraction F (sixth fraction) (320 mg) was shown by GC/MS to contain the major metabolite seen in chemogroup 1 and was subjected to normal phase HPLC conditions utilizing a hexane to 25% 1-chlorobutane gradient over 40 min on a preparative silica column to afford (in retention time order) compound **2** (4 mg) and anverene E (**9**, 30 mg).

*Anverene* B (**6**): clear oil, [α]${}_{D}^{25}$ −90 (*c* 0.1, CH_3_OH); UV (CH_3_OH) λ_max_ (log ε) 210 nm (3.85); IR υ (thin film): 3085, 2988, 2940, 1668, 1620, 1423, 976, 958, 740, 613 cm^−1^; ^1^H and ^13^C NMR data, see Table 1; 5 eV HRNCIMS (CH_4_) m/z 472.7477 [M − H]^−^ (calcd. for C_10_H_12_Br_3_Cl_3_, 472.7482). 

*Anverene C* (**7**): clear oil, [α]${}_{D}^{25}$ −80 (*c* 0.1, CH_3_OH); UV (CH_3_OH) λ_max_ (log ε) 205 nm (3.51); IR υ (thin film): 2937, 2855, 1665, 1465, 1393, 1384, 1379, 698, 643 cm^−1^; ^1^H and ^13^C NMR data, see Table 2; 5 eV HRNCIMS (CH_4_) m/z 440.8029 [M − H]^−^ (calcd. for C_10_H_15_Br_3_Cl_2_, 440.8028). 

*Anverene D (***8***):* clear oil, [α]${}_{D}^{25}$ −50 (*c* 0.04, CH_3_OH); UV (CH_3_OH) λ_max_ (log ε) 205 nm (3.59); IR υ (thin film): 2921 2861, 1638, 1385, 821, 604 cm^−1^; ^1^H and ^13^C NMR data, see Table 2; 5 eV HRNCIMS (CH_4_) m/z 396.8535 [M − H]^−^ (calcd. for C_10_H_15_Br_2_Cl_3_, 396.8533).

*Anverene E* (**9**): clear oil, [α]${}_{D}^{25}$ +90 (*c* 0.1, CH_3_OH); UV (CH_3_OH) λ_max_ (log ε) 235 nm (3.75); IR υ (thin film): 2983, 2933, 2881, 1678, 1628, 1465, 1448, 1092, 1089, 821, 673 cm^−1^; ^1^H and ^13^C NMR data, see Table 3; 5 eV HRNCIMS (CH_4_) m/z 376.8705 [M + Br]^−^ (calcd. for C_10_H_15_Br_2_Cl_3_, 376.8716).

### 4.4. Clonogenic Survival Assay

HeLa cells were seeded into 96 well plates at a concentration of 200 cells/mL in Dulbecco’s Modified Eagle’s Medium (DMEM) supplemented with 10% bovine serum. Compounds **1**–**9** were added to the medium 24 hours after seeding at concentrations of 30 µM, 15 µM, 10 µM, 3 µM, 1 µM, and 0.5 µM respectively, and the cells were allowed to grow for 5 days. The cells were fixed with a 10% methanol, 10% acetic acid solution (in water) for 15 min at room temperature and stained with 1% crystal violet (in methanol) for 5 min. Excess dye was removed with water and the plates were allowed to dry at room temperature overnight. Cells were de-stained with Sorenson’s buffer (0.1 M sodium citrate, 50% ethanol). The colorimetric intensity of each solution was quantified using Gen5 software on a Synergy 2 (BioTek, Winooksi, VT) plate reader (OD at 595 nm). The IC_50_ for each compound was calculated with error based on three independent experiments.

## Figures and Tables

**Table 1:** NMR data for anverene B (6) ^a^.

Pos	*δ*_C_^b^	*δ*_H_^c^	HMBC
1	110.3	6.56 (1H, d, 13.6)	2, 3, 4
2	138.8	6.41 (1H, d, 13.6)	1, 3, 4, 5, 10
3	70.9		
4	59.3	4.60 (1H, d, 9.5)	2, 3, 5, 6, 10
5	131.1	6.22 (1H, dd, 15.3, 9.6)	3, 4, 7
6	132.8	6.00 (1H, d, 15.3)	4, 7, 8, 9
7	68.8		
8a	37.2	3.83 (1H, d, 11.0)	6, 7, 9
b		3.87 (1H, d, 11.0)	6, 7, 9
9a	49.6	3.91 (1H, d, 11.7)	6, 7, 8
b		3.98 (1H, d, 11.7)	6, 7, 8
10	25.9	1.81 (3H, s)	1, 2, 3, 4

^a^ CDCl_3_, ppm; ^b^ 125 MHz; ^c^ 500 MHz, (integration, multiplicity, *J* (Hz)).

**Table 2:** NMR data for anverenes C (**7**) and D (**8**) ^a^.

Pos	*δ*_C_^b^	Anverene C (7) *δ*_H_^c^	HMBC	*δ*_C_^b^	Anverene D (8) *δ*_H_^c^	HMBC
1	45.1	5.68 (1H, d, 9.2)	2, 3, 10	66.6	6.40 (1H, d, 9.6)	3
2	73.4	4.80 (1H, d, 9.3)	1, 3, 4, 10	128.0	6.03 (1H, d, 9.3)	4, 10
3	134.0			138.2		
4	130.0	5.83 (1H, t, 7.2)	2, 5, 6, 10	57.8	4.81 (1H, br, dd, 11.3)	2, 3, 5, 6, 10
5a	33.5	2.53 (1H, ddd, 15.1, 10.8, 7.3)	3, 4, 6	41.0	2.13 (1H, br, td, 13.2)	6
b		3.15 (1H, dd, 15.2, 6.9)	3, 4, 7		2.88 (1H, br, td, 13.2)	3, 4, 7
6	68.8	4.03 (1H, dd, 10.8, 2.0)	4, 5, 7, 8, 9	62.7	4.43 (1H, br, dd, 11.3)	4, 5, 7, 8, 9
7	70.5			70.8		
8	27.7	1.80 (3H, s)	6, 7, 9	28.1	1.71 (3H, s)	6, 7, 9
9	33.6	1.93 (3H, s)	6, 7, 8	32.8	1.84 (3H, s)	6, 7, 8
10	11.2	1.76 (3H, s)	2, 3, 4	13.9	1.94 (3H, s)	2, 3, 4

^a^ CDCl_3_, ppm; ^b^ 125 MHz; ^c^ 500 MHz, (integration, multiplicity, *J* (Hz)); br, broad signals.

**Table 3:** NMR data for anverene E (**9**) ^a^.

	*δ*_C_^b^	*δ*_H_^c^	HMBC
1	108.2	6.56 (1H, d, 14.1)	2, 3
2	136.2	6.73 (1H, d, 14.1)	1, 4, 10
3	143.2		
4	72.0	4.68 (1H, dd, 8.3, 2.7)	3, 8, 10
5β	37.3	2.30 (1H, ddd, 14.8, 8.4, 3.4)	4
α		2.42 (1H, ov, m)	
6	58.0	4.52 (1H, t, 3.6)	
7	66.4		
8β	71.5	3.65 (1H, d, 11.8)	4, 6, 9
α		4.05 (1H, d, 11.8)	4, 6, 9
9	27.1	1.80 (3H, s)	6, 7, 8
10a	116.8	5.26 (1H, s)	2, 3, 4
b		5.28 (1H, s)	1, 2, 3, 4

^a^ CDCl_3_, ppm; ^b^ 125 MHz; ^c^ 500 MHz, (integration, multiplicity, *J* (Hz)); br –broad signals; m – complex multiplet.

**Table 4:** In vitro cytotoxicity of **1**–**9** towards HeLa cells.

Compound	IC_50_ (µM)	Standard Error
Anverene A (**1**)	8.36	±1.75
Compound **2**	1.14	±0.06
Oregonene A (**3**)	1.3	±0.04
Compound **4**	12.49	±1.47
Compound **5**	12.76	±3.43
Anverene B (**6**)	10.53	±0.83
Anverene C (**7**)	1.19	±0.04
Anverene D (**8**)	5.84	±1.08
Anverene E (**9**)	9.81	±1.66

## Supplementary Materials

The following are available online at https://www.mdpi.com/1660-3397/17/4/230/s1, Figure S1: Anverene B ^1^H NMR spectrum (500 MHz, CDCl_3_), Figure S2: Anverene B ^13^C NMR spectrum (125 MHz, CDCl_3_), Figure S3: Anverene B COSY spectrum (500 MHz, CDCl_3_), Figure S4: Anverene B HSQC spectrum (500 MHz, CDCl_3_), Figure S5: Anverene B HMBC spectrum (500 MHz, CDCl_3_), Figure S6: Anverene B D HRNCIMS (-), Figure S7: Anverene C ^1^H NMR spectrum (500 MHz, CDCl_3_), Figure S8: Anverene C ^13^C NMR spectrum (125 MHz, CDCl_3_), Figure S9: Anverene C COSY spectrum (500 MHz, CDCl_3_), Figure S10: Anverene C HSQC spectrum (500 MHz, CDCl_3_), Figure S11: Anverene C HMBC spectrum (500 MHz, CDCl_3_), Figure S12: Anverene C 1D NOESY spectrum (600 MHz, CDCl_3_), Figure S13: Anverene C HRNCIMS (-), Figure S14: Anverene D ^1^H NMR spectrum (500 MHz, CDCl_3_), Figure S15: Anverene D ^13^C NMR spectrum (125 MHz, CDCl_3_), Figure S16: Anverene D COSY spectrum (500 MHz, CDCl_3_), Figure S17: Anverene D HSQC spectrum (500 MHz, CDCl_3_), Figure S18: Anverene D HMBC spectrum (500 MHz, CDCl_3_), Figure S19: Anverene C 1D NOESY spectrum (600 MHz, CDCl_3_), Figure S20: Anverene D HRNCIMS (-), Figure S21: Anverene E ^1^H NMR spectrum (500 MHz, CDCl_3_), Figure S22: Anverene E ^13^C NMR spectrum (125 MHz, CDCl_3_), Figure S23: Anverene E COSY spectrum (500 MHz, CDCl_3_), Figure S24: Anverene E HSQC spectrum (500 MHz, CDCl_3_), Figure S25: Anverene E HMBC spectrum (500 MHz, CDCl_3_), Figure S26: Anverene E NOESY spectrum (500 MHz, CDCl_3_), Figure S27: Anverene E HRNCIMS (-), Figure S28: GC/MS identification of Anverene E in crude extract, Table S1: Reported and predicted ^13^C NMR shifts of relevant compounds or theoretical alternate regio-isomers.
