# Supplementary material for: Anverenes B–E, New Polyhalogenated Monoterpenes from the Antarctic Red Alga Plocamium cartilagineum
Source: Mar Drugs. 2019 Apr 17;17(4):230. doi: 10.3390/md17040230 (PMC6521118; doi:10.3390/md17040230)
Supplement: Supplementary file 1 [file marinedrugs-17-00230-s001.pdf]

marine drugs

Supplementary Information

Anverenes B-E, New polyhalogenated monoterpenes from the Antarctic red alga *Plocamium cartilagineum*

Andrew J. Shilling [1], Jacqueline L. von Salm [1], Anthony R. Sanchez [2], Younghoon Kee [2], Charles D. Amsler [3], James B. McClintock [3], and Bill J. Baker [1,*]

[1] Department of Chemistry, University of South Florida, 4202 E. Fowler Ave., CHE205, Tampa, Florida 33620, United States; bjbaker@usf.edu

[2] Department of Cell Biology, Microbiology, and Molecular Biology, University of South Florida, 4202 E. Fowler Ave., ISA2015, Tampa, Florida 33620, United States; ykee@usf.edu

[3] Department of Biology, University of Alabama at Birmingham, Birmingham, AL 35294, United States; amsler@uab.edu (C.D.A.); mcclinto@uab.edu (J.B.M.)

Page

Figure S1: Anverene B ^{1}H NMR spectrum (500 MHz, $CDCl_3$). 2

Figure S2: Anverene B ^{13}C NMR spectrum (125 MHz, $CDCl_3$). 2

Figure S3: Anverene B COSY spectrum (500 MHz, $CDCl_3$). 3

Figure S4: Anverene B HSQC spectrum (500 MHz, $CDCl_3$). 3

Figure S5: Anverene B HMBC spectrum (500 MHz, $CDCl_3$). 4

Figure S6: Anverene B D HRNCIMS (-). 4

Figure S7: Anverene C ^{1}H NMR spectrum (500 MHz, $CDCl_3$). 5

Figure S8: Anverene C ^{13}C NMR spectrum (125 MHz, $CDCl_3$). 5

Figure S9: Anverene C COSY spectrum (500 MHz, $CDCl_3$). 6

Figure S10: Anverene C HSQC spectrum (500 MHz, $CDCl_3$). 6

Figure S11: Anverene C HMBC spectrum (500 MHz, $CDCl_3$). 7

Figure S12: Anverene C 1D NOESY spectrum (600 MHz, $CDCl_3$). 7

Figure S13: Anverene C HRNCIMS (-). 8

Figure S14: Anverene D ^{1}H NMR spectrum (500 MHz, $CDCl_3$). 8

Figure S15: Anverene D ^{13}C NMR spectrum (125 MHz, $CDCl_3$). 9

Figure S16: Anverene D COSY spectrum (500 MHz, $CDCl_3$). 9

Figure S17: Anverene D HSQC spectrum (500 MHz, $CDCl_3$). 10

Figure S18: Anverene D HMBC spectrum (500 MHz, $CDCl_3$). 10

Figure S19: Anverene C 1D NOESY spectrum (600 MHz, $CDCl_3$). 11

Figure S20: Anverene D HRNCIMS (-). 11

Figure S21: Anverene E ^{1}H NMR spectrum (500 MHz, $CDCl_3$). 12

Figure S22: Anverene E ^{13}C NMR spectrum (125 MHz, $CDCl_3$). 12

Figure S23: Anverene E COSY spectrum (500 MHz, $CDCl_3$). 13

Figure S24: Anverene E HSQC spectrum (500 MHz, $CDCl_3$). 13

Figure S25: Anverene E HMBC spectrum (500 MHz, $CDCl_3$). 14

Figure S26: Anverene E NOESY spectrum (500 MHz, $CDCl_3$). 14

Figure S27: Anverene E HRNCIMS (-). 15

Figure S28: GC/MS identification of Anverene E in crude extract 15

Table S1: Reported and predicted ^{13}C NMR shifts of relevant compounds or theoretical alternate regio-isomers 16-18

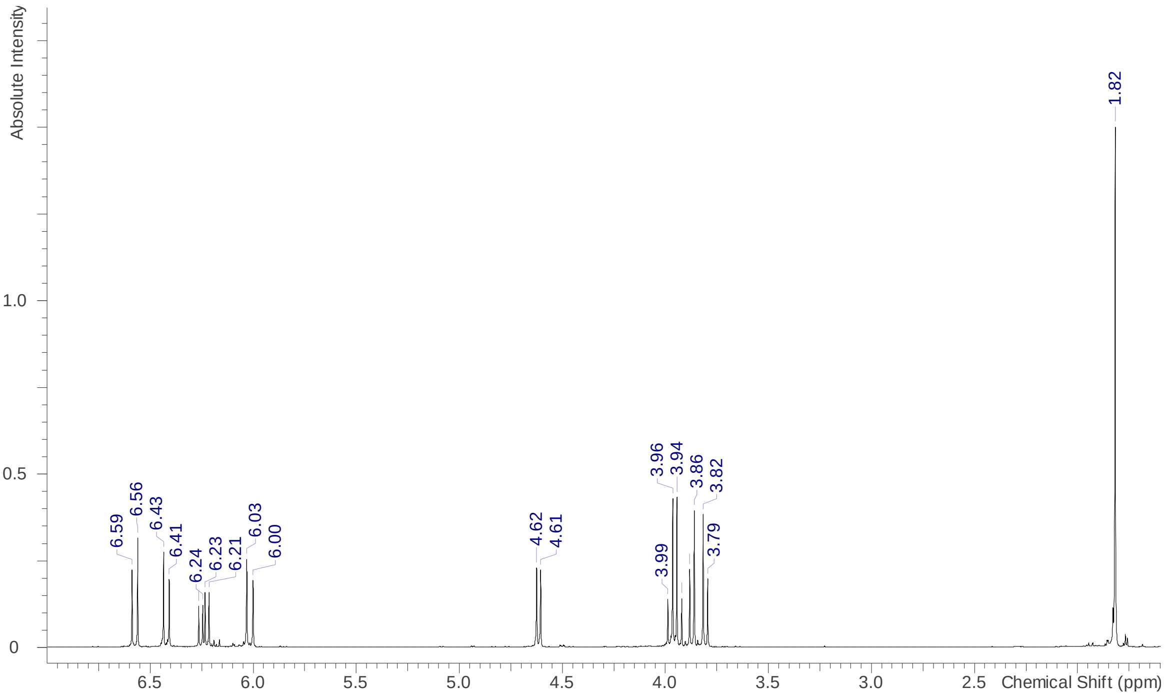

Figure S1: Anverene B ^{1}H NMR spectrum (500 MHz, $CDCl_3$).

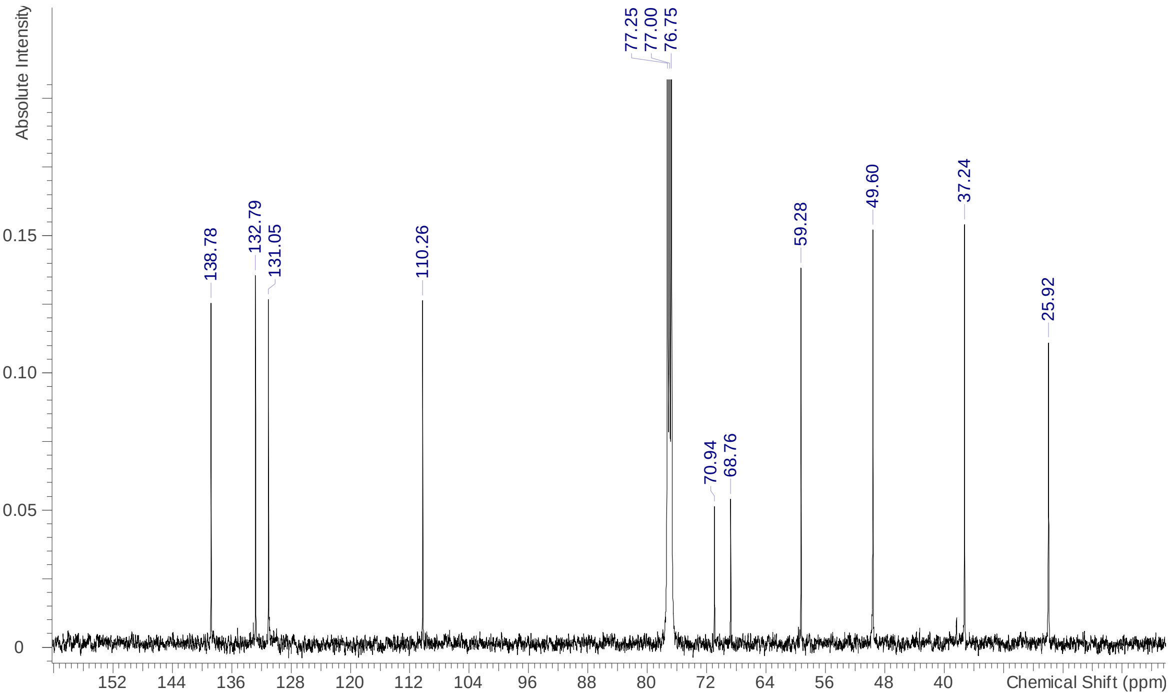

Figure S2: Anverene B ^{13}C NMR spectrum (125 MHz, $CDCl_3$).

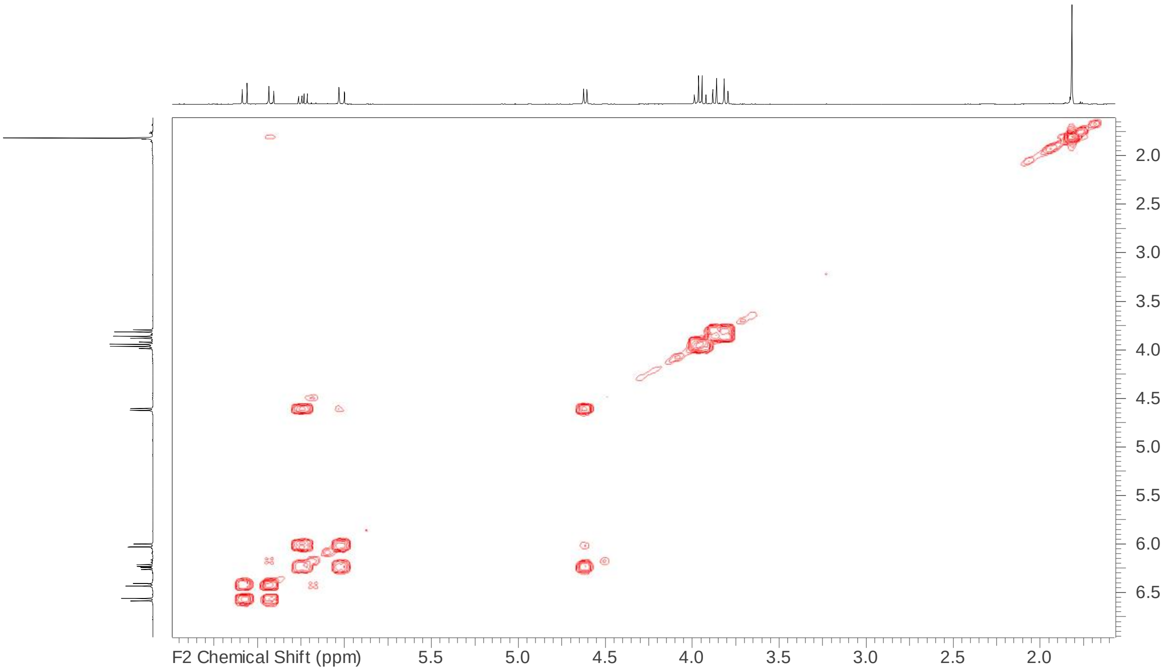

Figure S3: Anverene B COSY spectrum (500 MHz, $CDCl_3$).

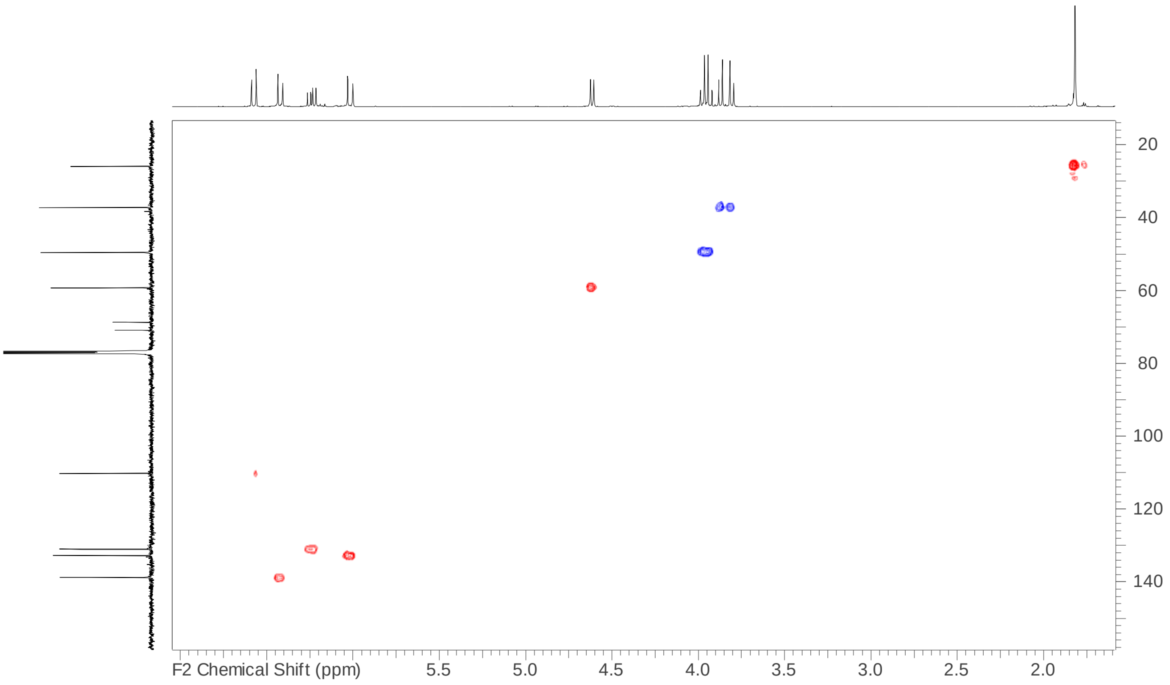

Figure S4: Anverene B HSQC spectrum (500 MHz, $CDCl_3$).

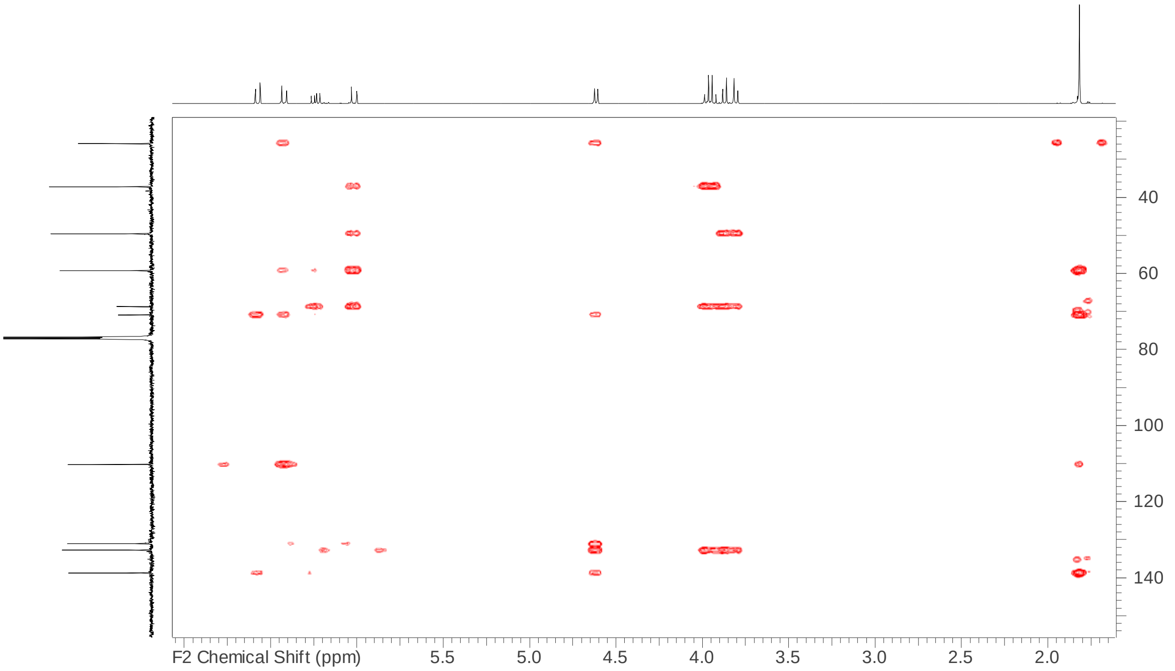

Figure S5: Anverene B HMBC spectrum (500 MHz, $CDCl_3$).

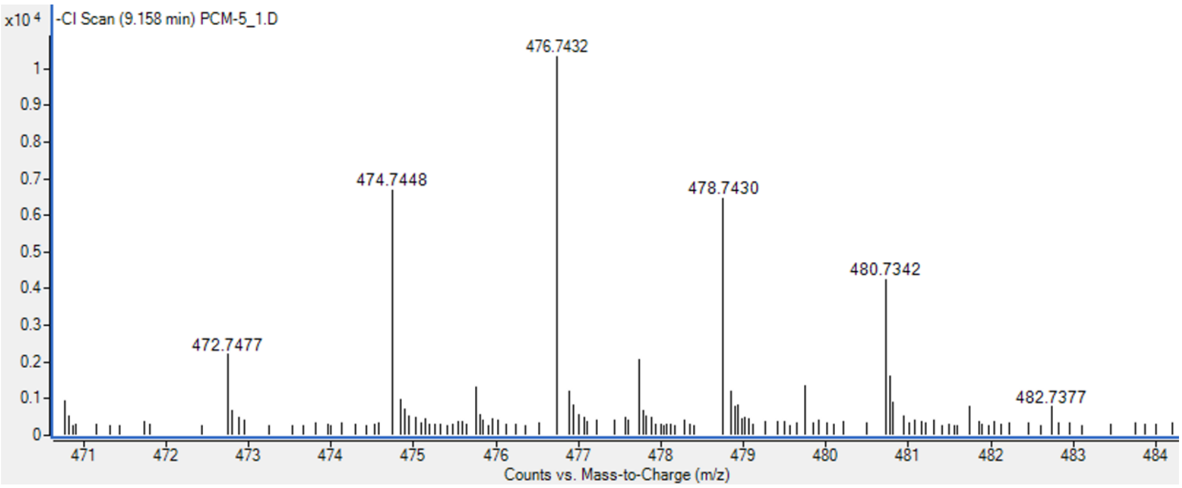

Formula	Species	Abundance (counts)	Ion Mass	Measured Mass	Error (ppm)	Error (mDa)
C10H12Br3Cl3	[M-H]-	2231.10	472.7482	472.7477	-1.058	-0.5

Figure S6: Anverene B HRNCIMS (-).

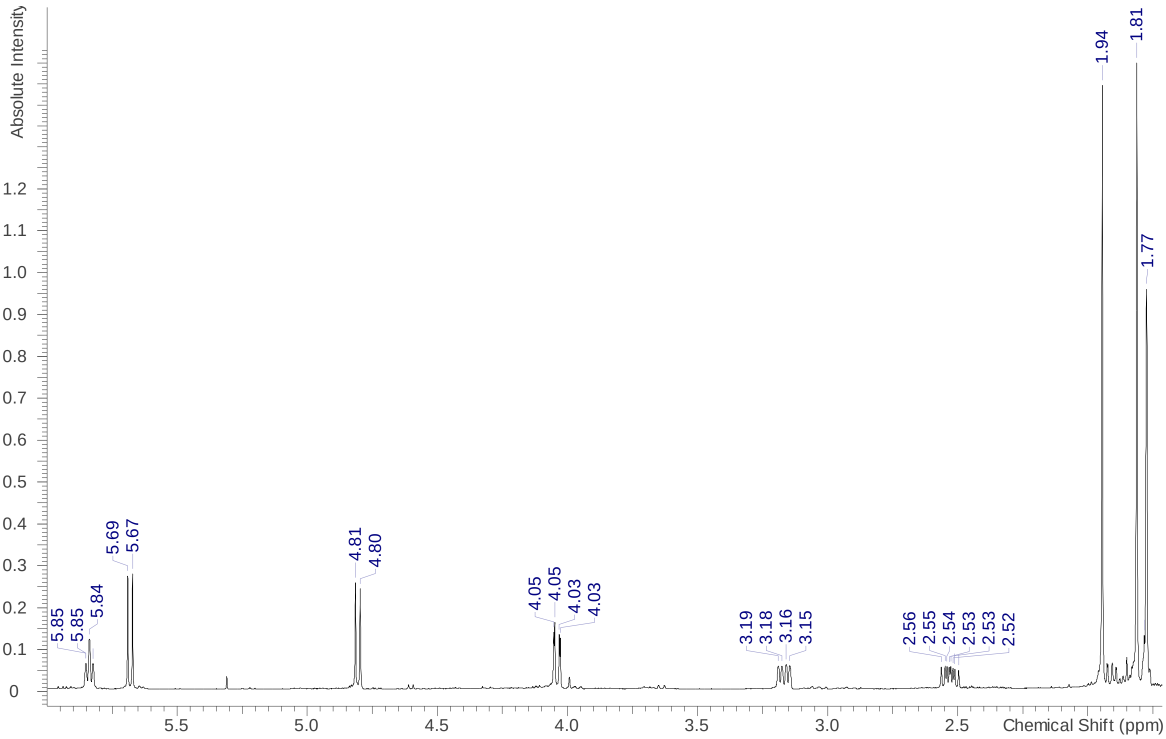

Figure S7: Anverene C ^{1}H NMR spectrum (500 MHz, $CDCl_3$).

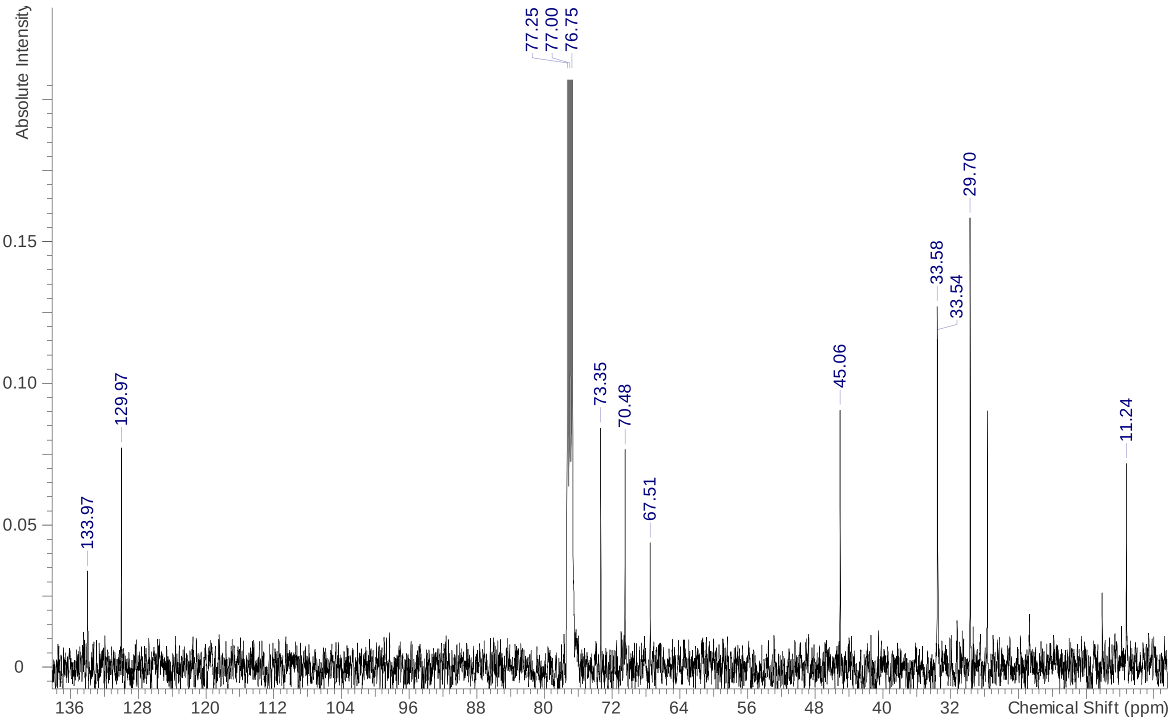

Figure S8: Anverene C ^{13}C NMR spectrum (125 MHz, $CDCl_3$).

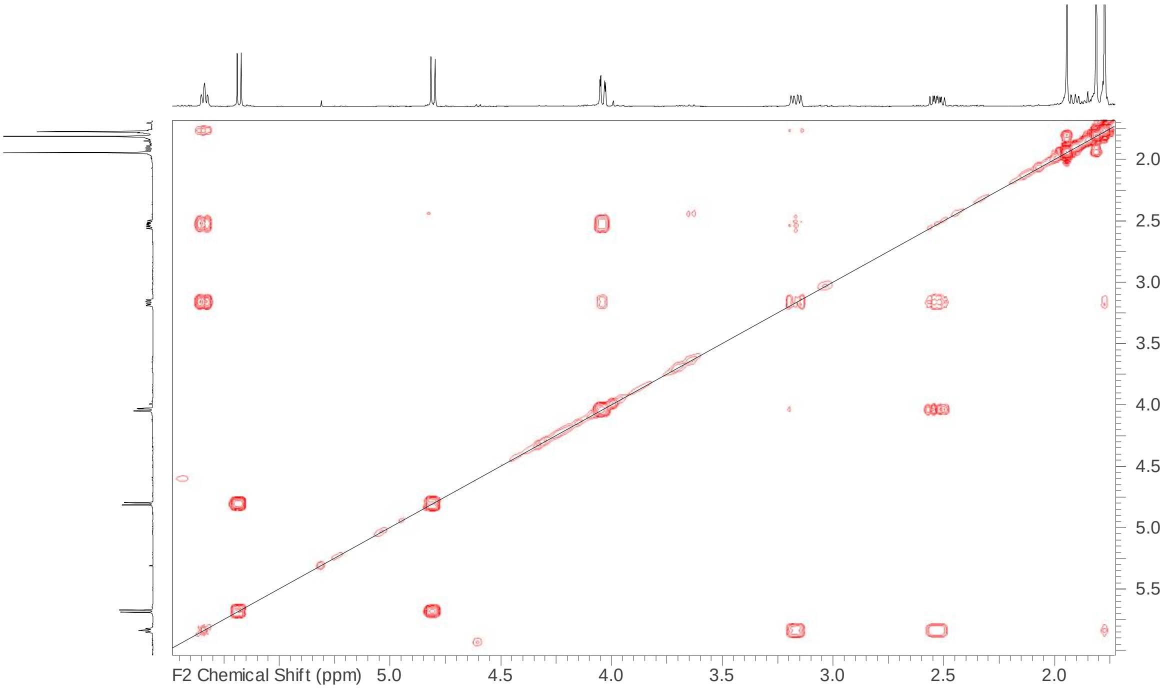

Figure S9: Anverene C COSY spectrum (500 MHz, $CDCl_3$).

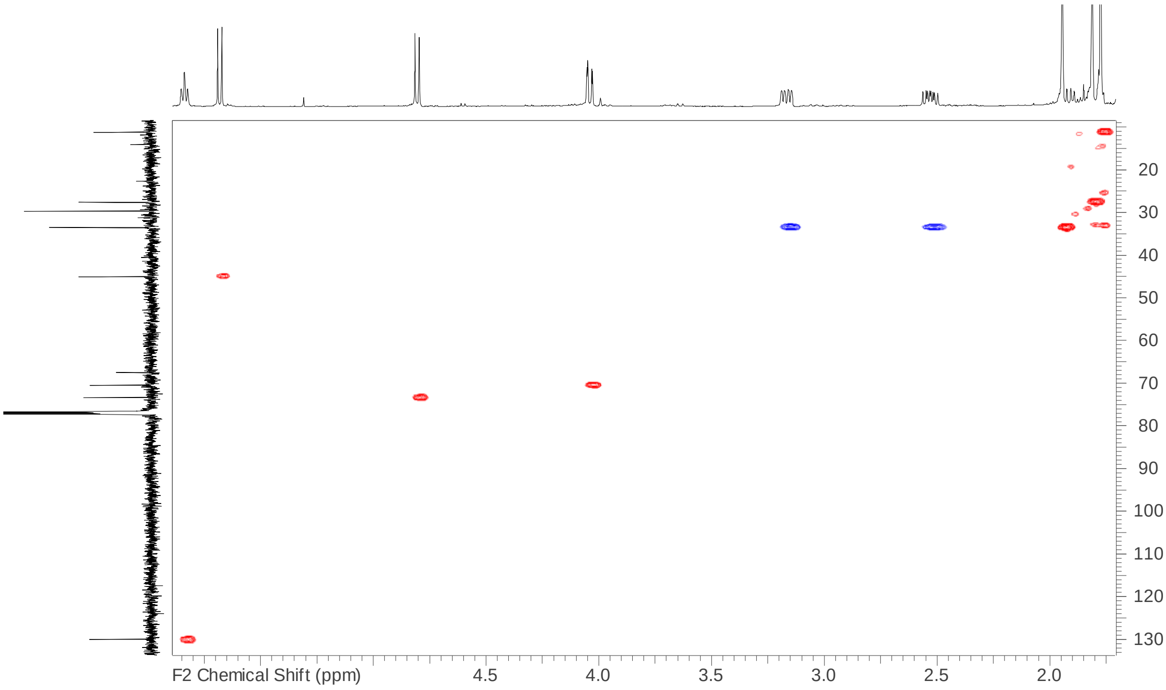

Figure S10 Anverene C HSQC spectrum (500 MHz, $CDCl_3$).

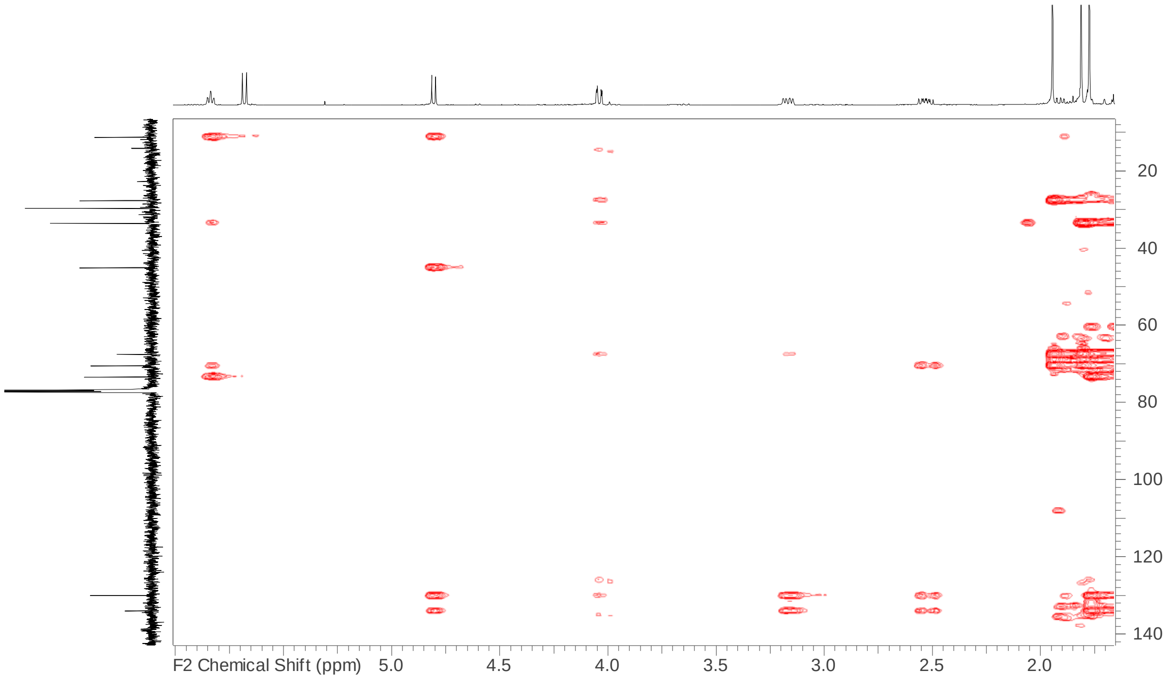

Figure S11: Anverene C HMBC spectrum (500 MHz, $CDCl_3$).

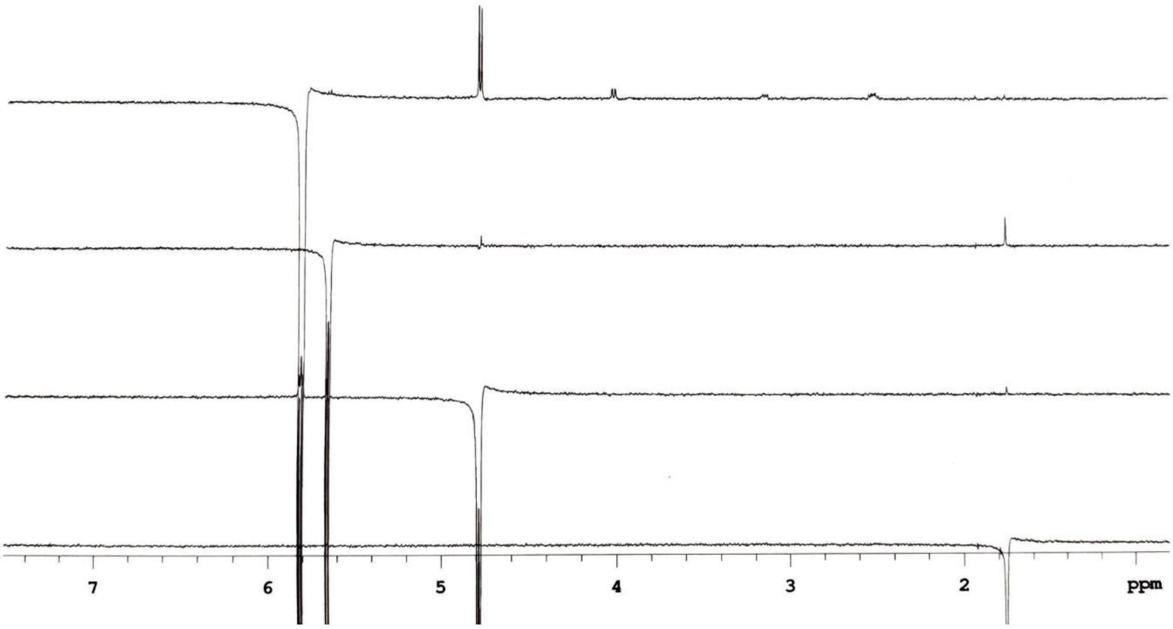

Figure S12: Anverene C 1D NOESY spectrum (600 MHz, $CDCl_3$).

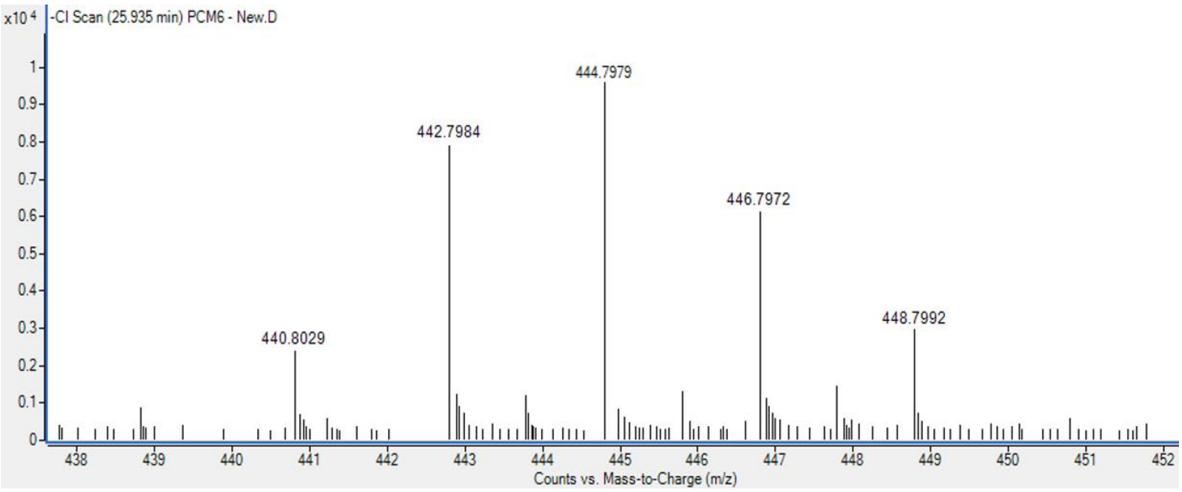

Formula	Species	Abundance (counts)	Ion Mass	Measured Mass	Error (ppm)	Error (mDa)
C10H15Br3Cl2	[M-H]-	2403.53	440.8028	440.8029	0.227	0.1

Figure S13: Anverene C HRNCIMS (-).

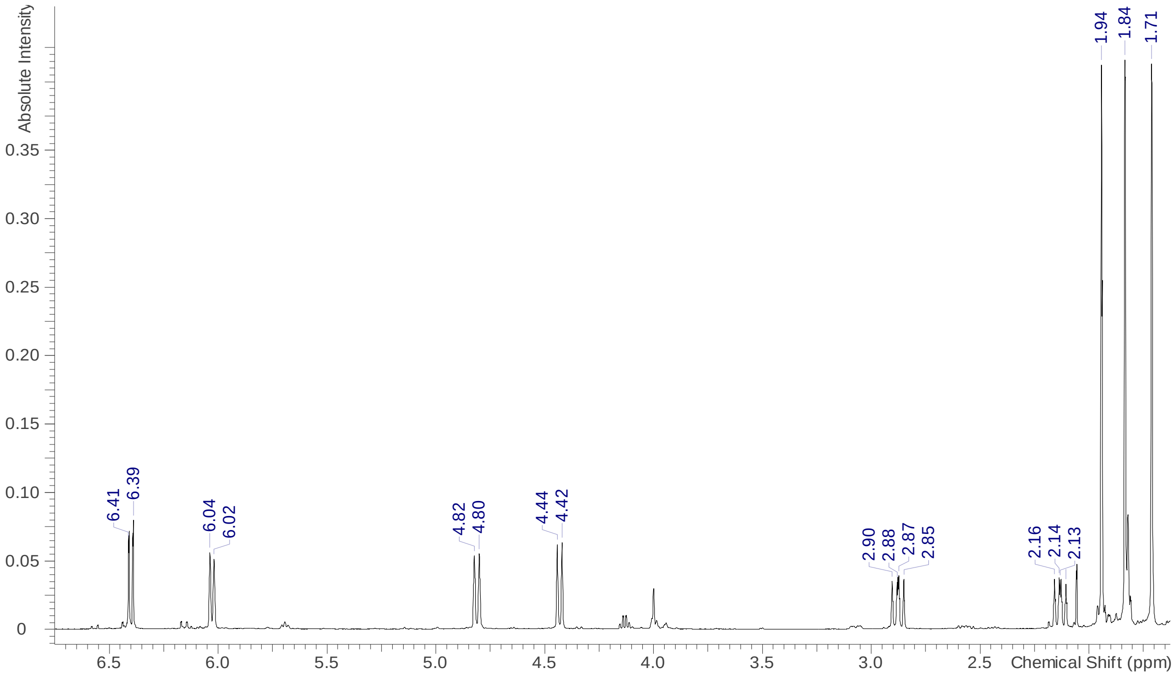

Figure S14: Anverene D ^{1}H NMR spectrum (500 MHz, $CDCl_3$).

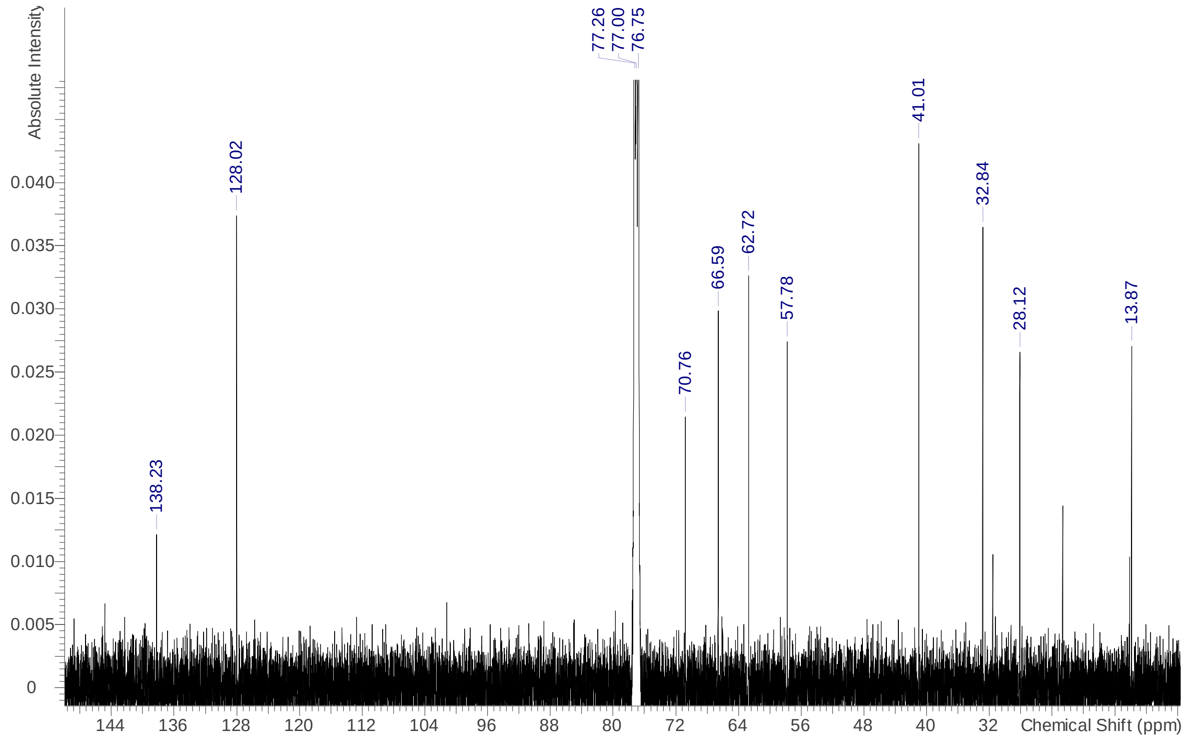

Figure S15: Anverene D ^{13}C NMR spectrum (125 MHz, $CDCl_3$).

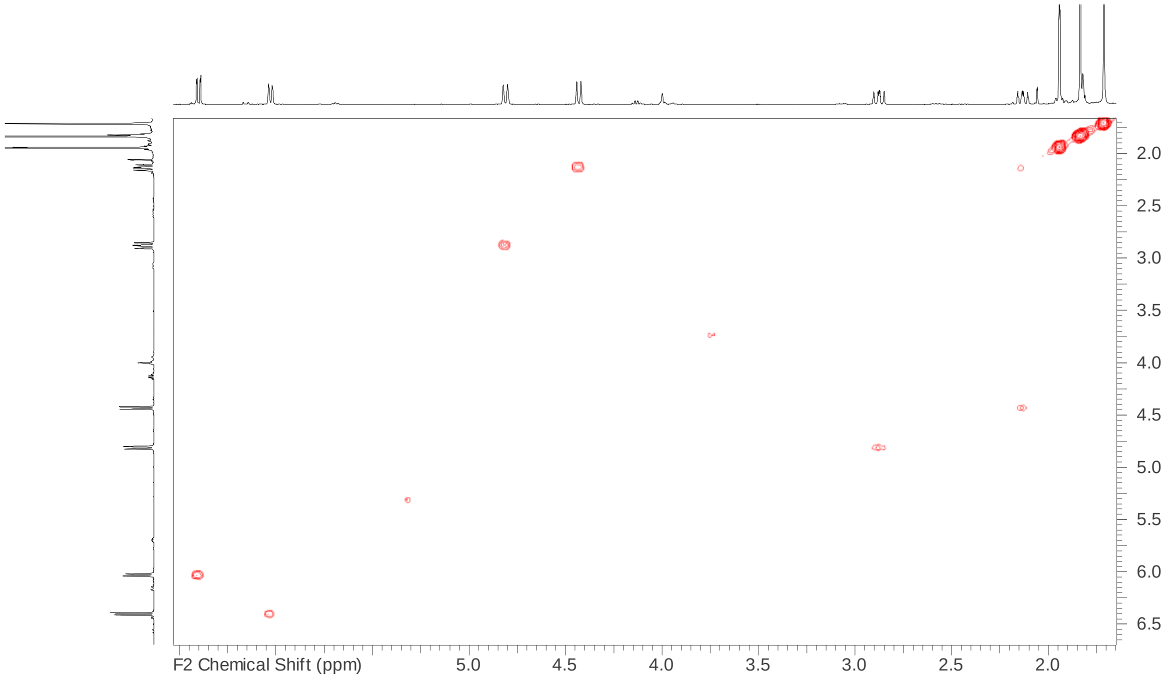

Figure S16: Anverene D COSY spectrum (500 MHz, $CDCl_3$).

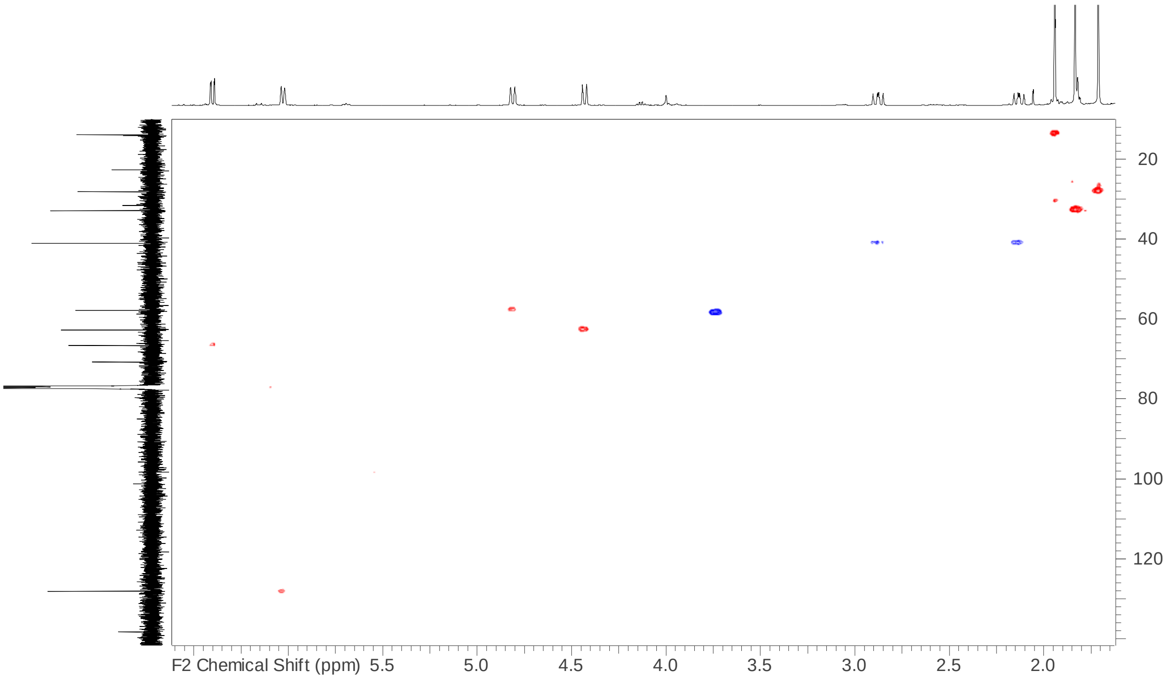

Figure S17: Anverene D HSQC spectrum (500 MHz, $CDCl_3$).

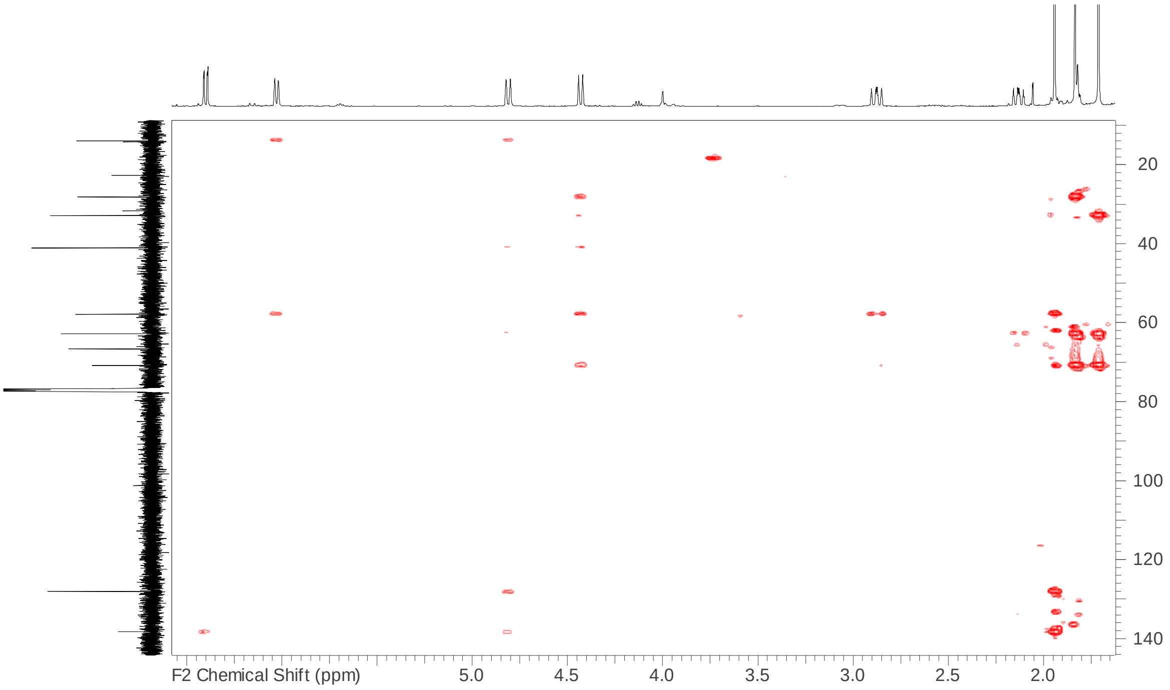

Figure S18: Anverene D HMBC spectrum (500 MHz, $CDCl_3$).

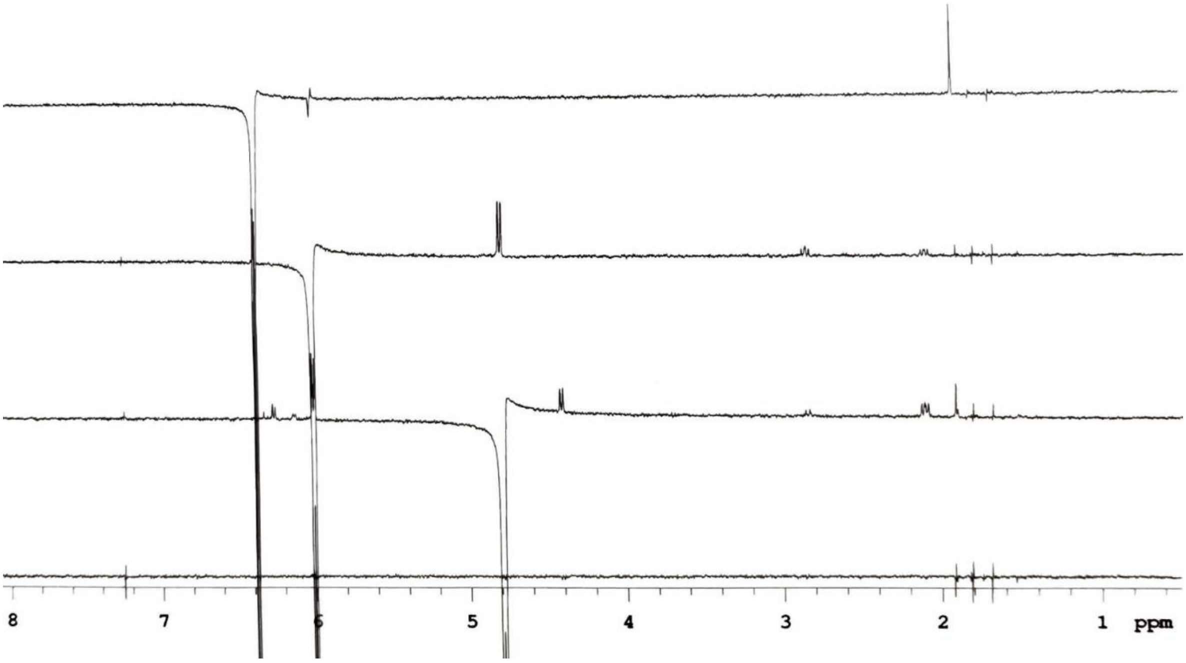

Figure S19: Anverene C 1D NOESY spectrum (600 MHz, $CDCl_3$).

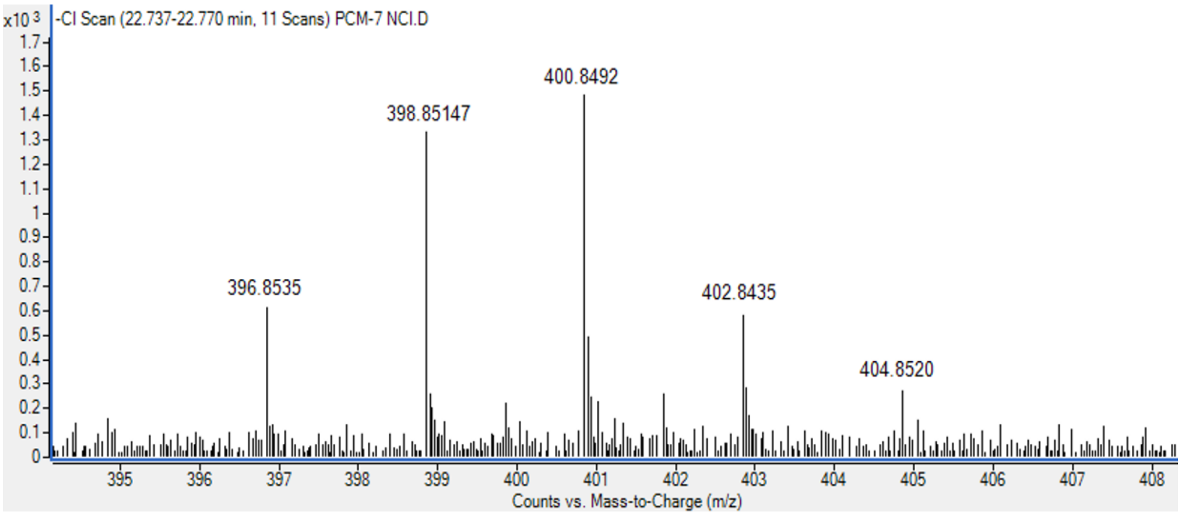

Formula	Species	Abundance (counts)	Ion Mass	Measured Mass	Error (ppm)	Error (mDa)
C10H15Br2Cl3	[M-H]-	610.34	396.8533	396.8535	0.504	0.2

Figure S20: Anverene D HRNCIMS (-).

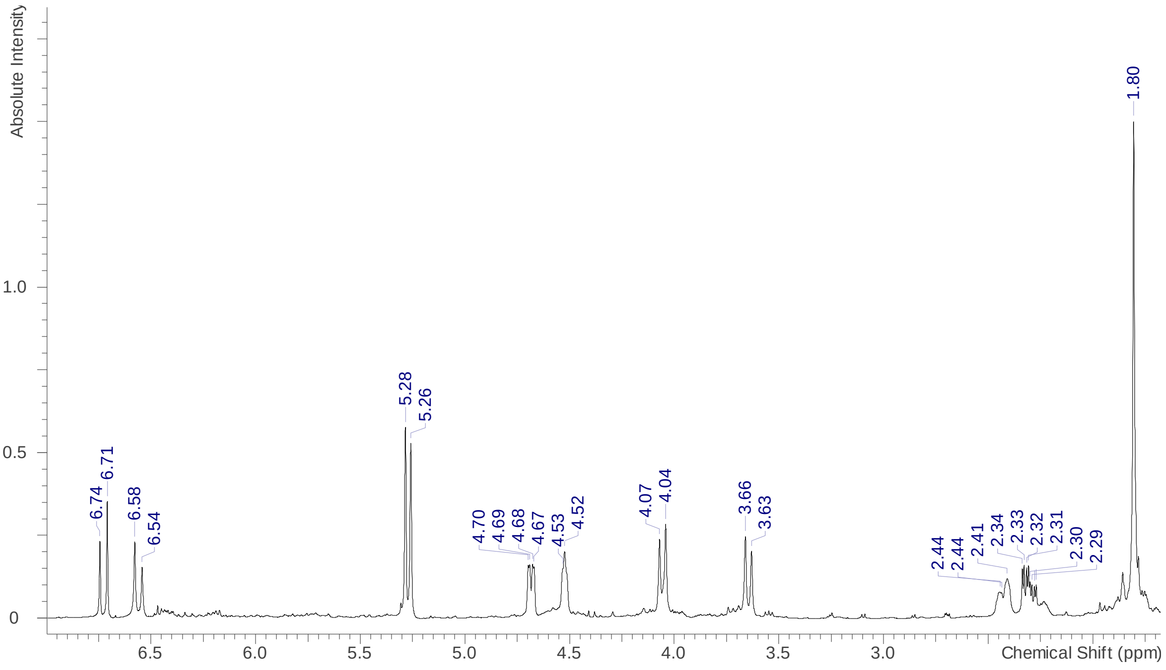

Figure S21: Anverene E ^{1}H NMR spectrum (500 MHz, $CDCl_3$).

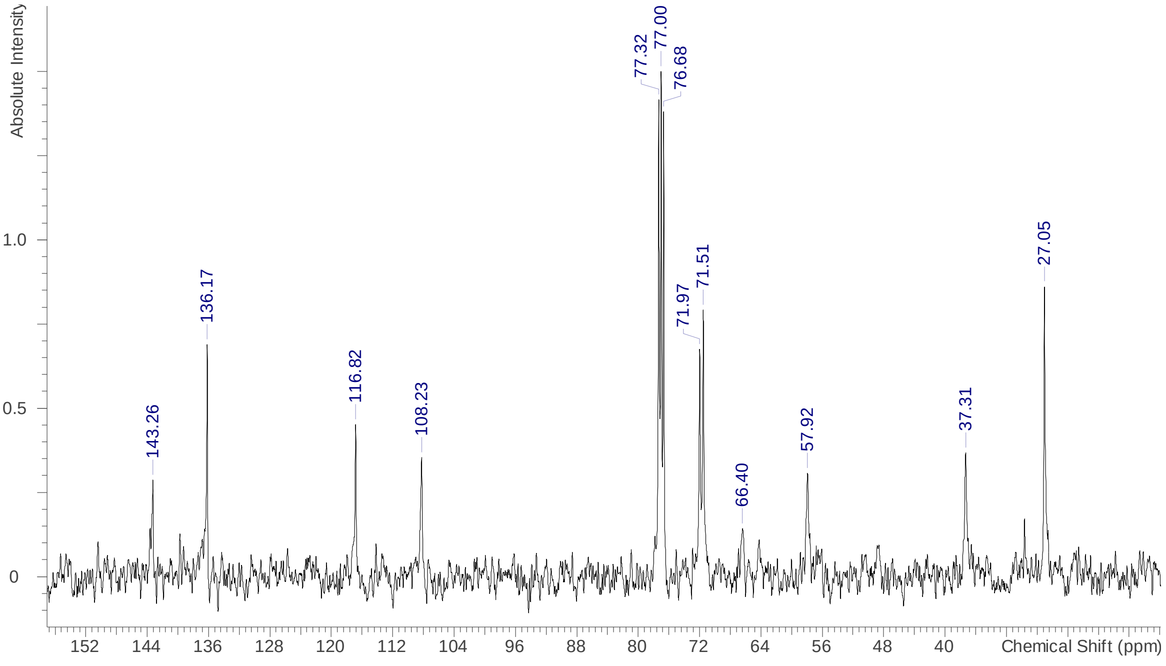

Figure S22: Anverene E ^{13}C NMR spectrum (125 MHz, $CDCl_3$).

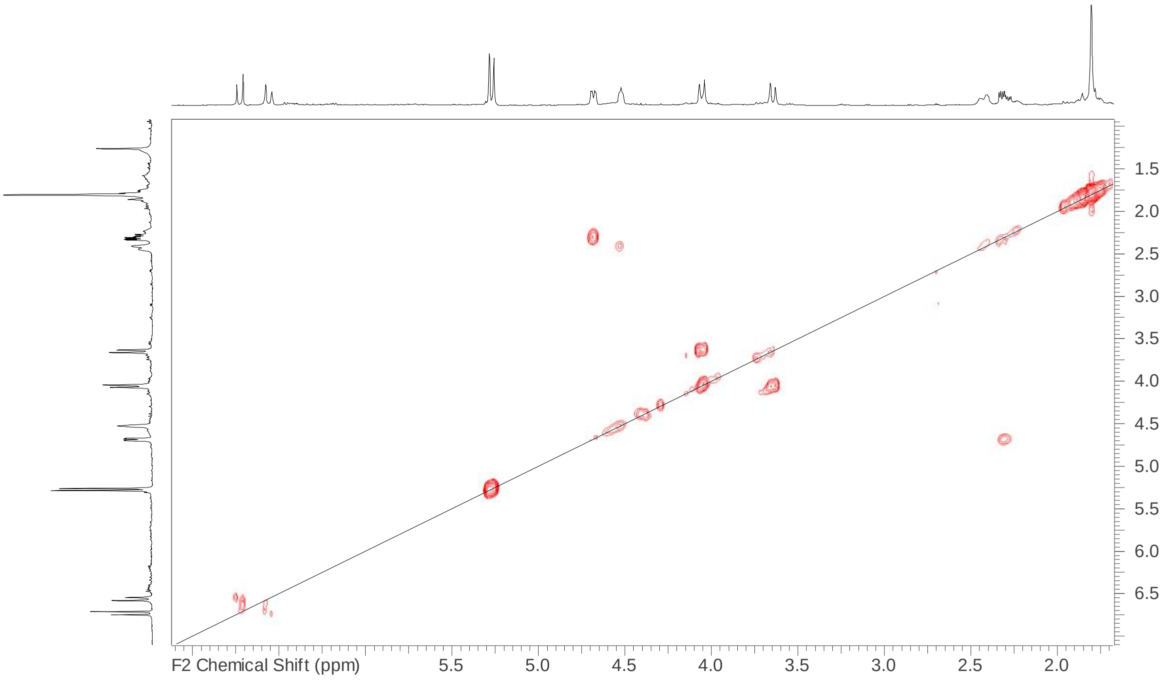

Figure S23: Anverene E COSY spectrum (500 MHz, $CDCl_3$).

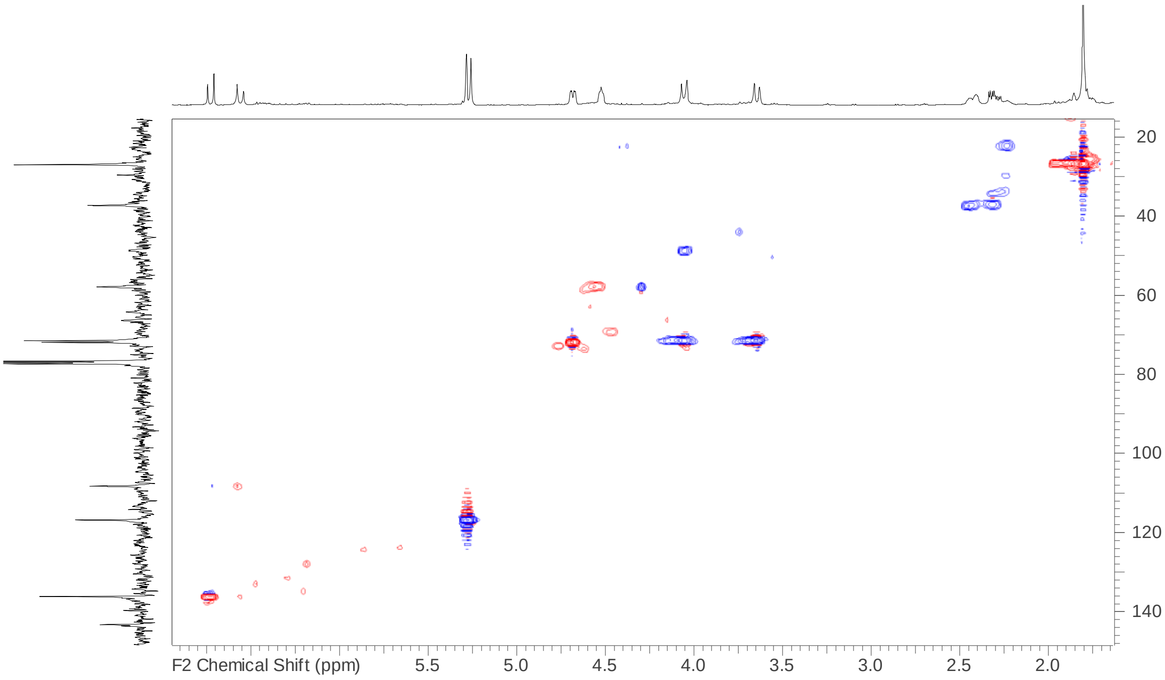

Figure S24: Anverene E HSQC spectrum (500 MHz, $CDCl_3$).

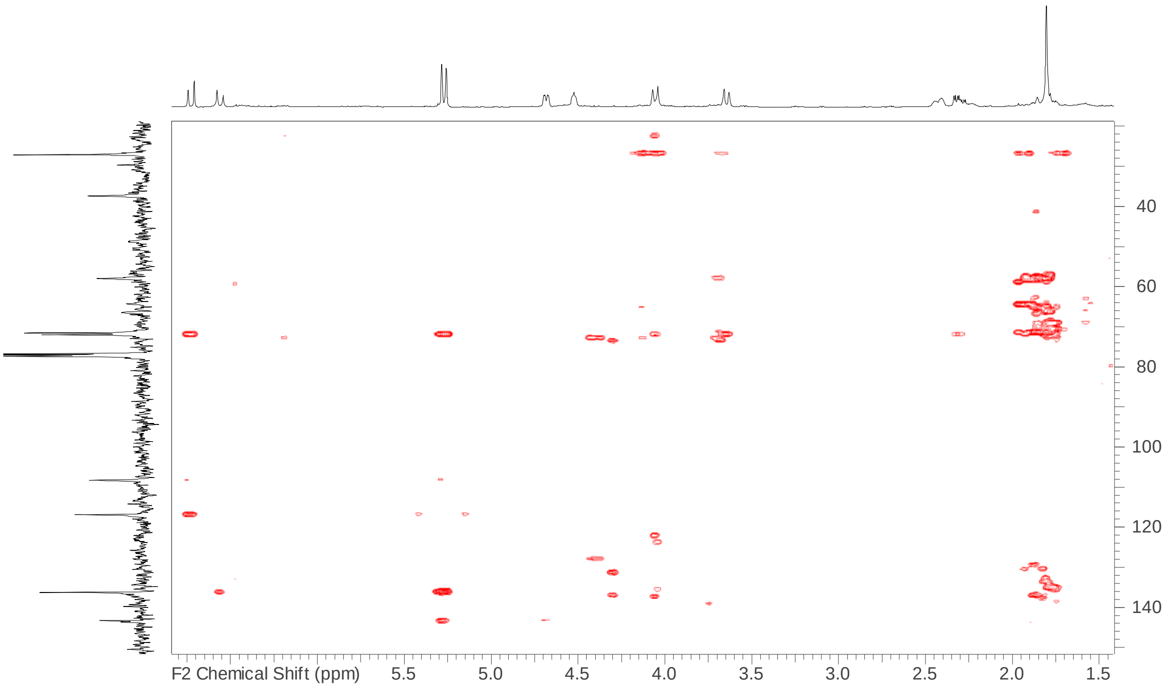

Figure S25: Anverene E HMBC spectrum (500 MHz, $CDCl_3$).

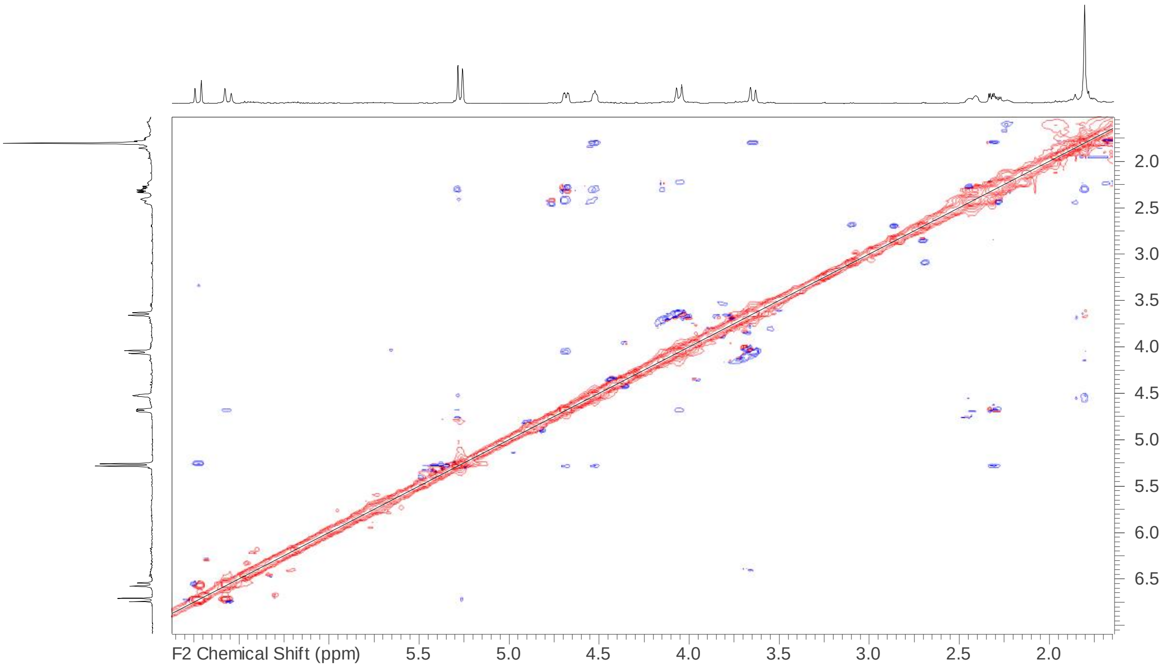

Figure S26: Anverene E NOESY spectrum (500 MHz, $CDCl_3$).

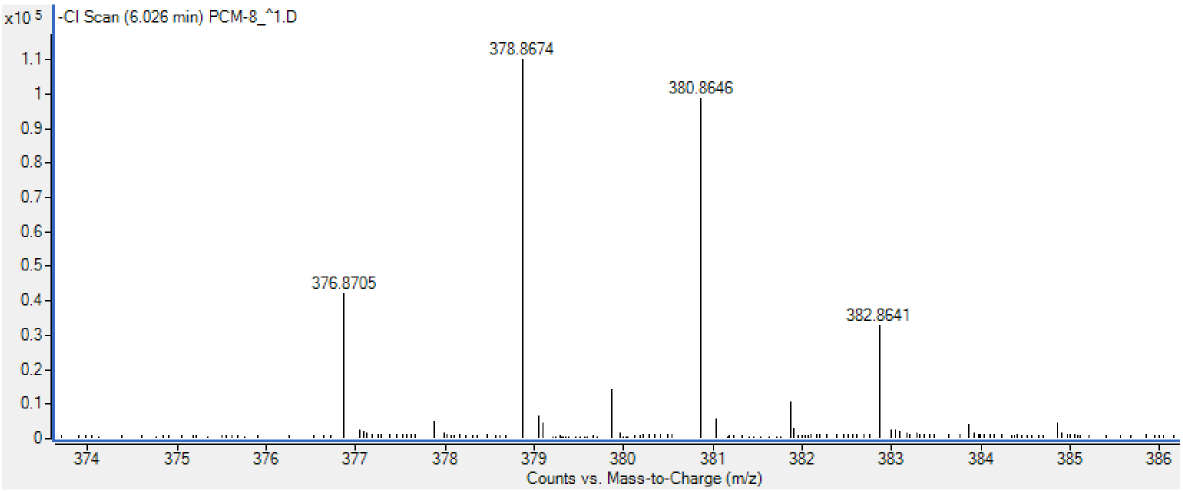

Formula	Species	Abundance (counts)	Ion Mass	Measured Mass	Error (ppm)	Error (mDa)
C10H13BrCl2O	[M+Br]-	42070.74	376.8716	376.8705	-2.918	-1.1

Figure S27: Anverene E HRNCIMS (-).

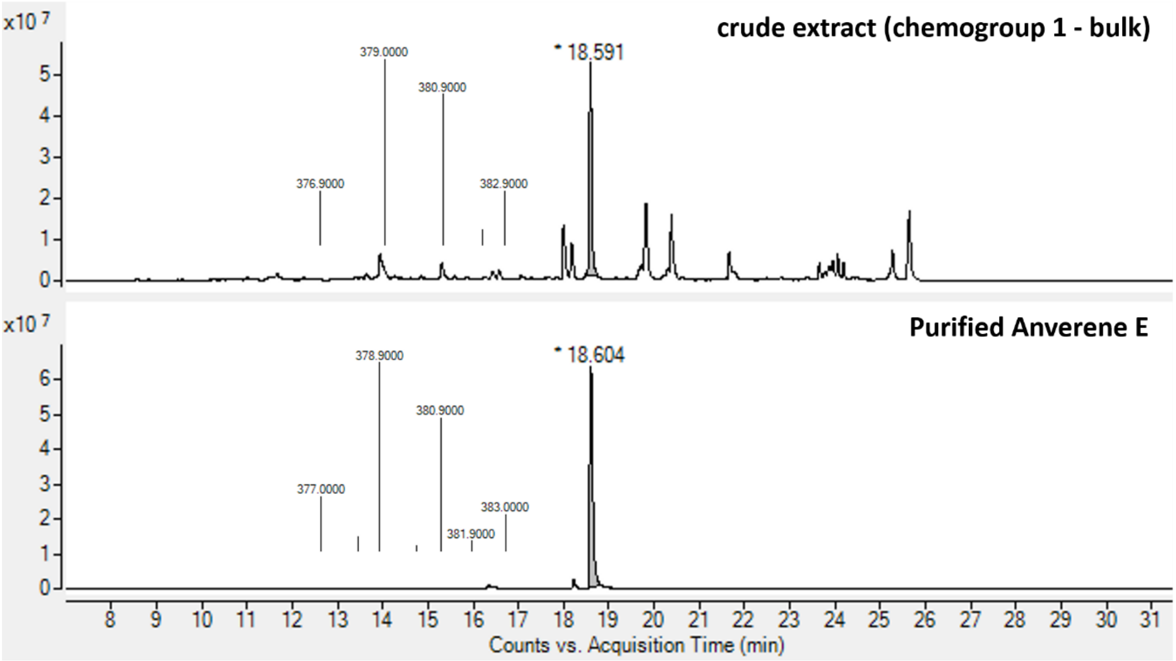

Figure S28: GC/MS identification of Anverene E in crude extract

Table S1: Reported and predicted ^{13}C NMR shifts of relevant compounds or possible regio-isomers

Compound	Reported ^{13}C Shifts	ChemDraw Chem NMR ^{13}C Estimations
**(1)** Anverene	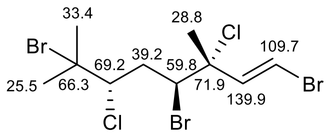	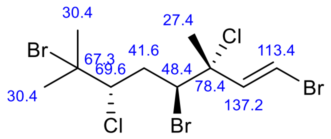
**(2)**	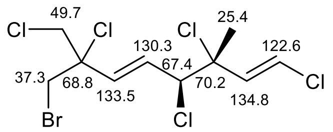	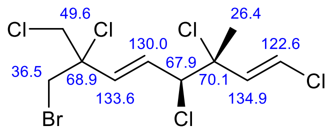
**(3)** Oregonene A	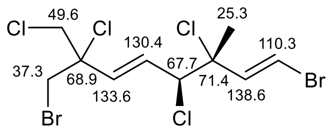	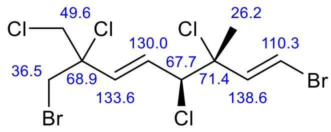
**(4)**	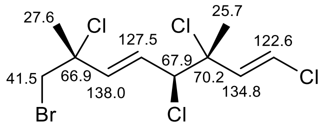	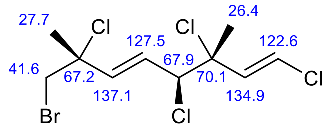
**(5)**	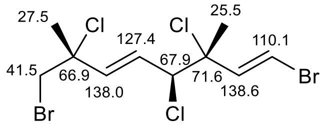	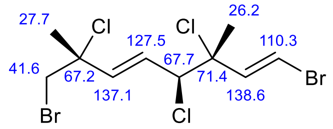
**(6)** Anverene B	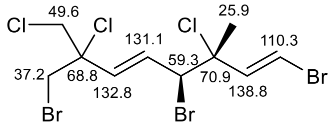	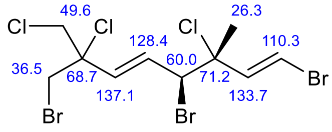
Theoretical Regio-Isomer Of Anverene B		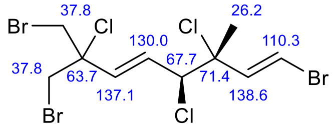
Theoretical Regio-Isomer Of Anverene B		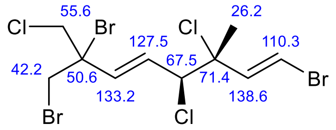
Theoretical Regio-Isomer Of Anverene B		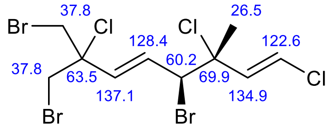

Theoretical Regio-Isomer Of Anverene B		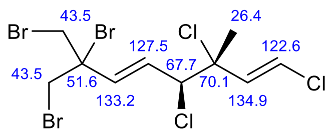
Theoretical Regio-Isomer Of Anverene B		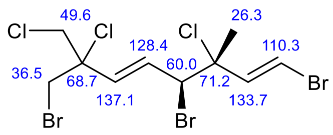
(**7**) Anverene C	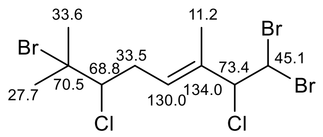	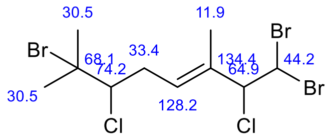
(**10**)	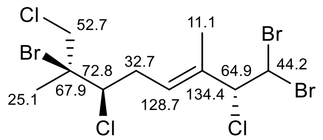	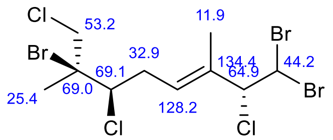
Theoretical Regio-Isomer Of Anverene C		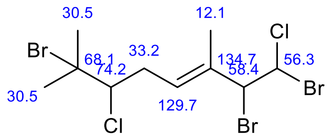
Theoretical Regio-Isomer Of Anverene C		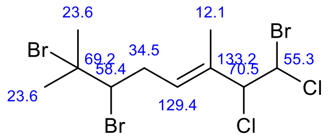
Theoretical Regio-Isomer Of Anverene C		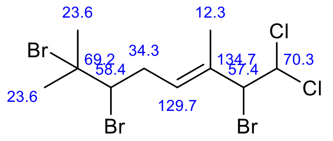
Theoretical Regio-Isomer Of Anverene C		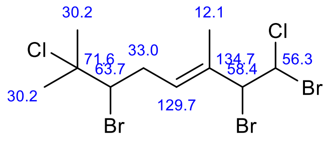
Theoretical Regio-Isomer Of Anverene C		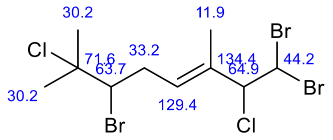
(**8**) Anverene D	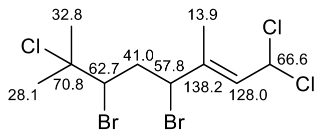	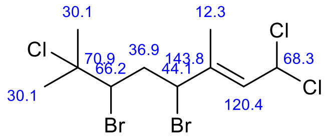

(**11**)<br>Plocoralide B	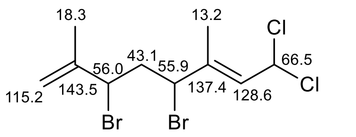	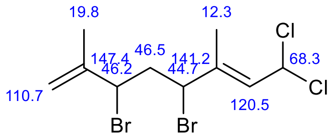
Theoretical<br>Regio-Isomer<br>Of Anverene D		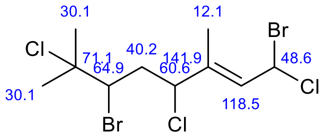
Theoretical<br>Regio-Isomer<br>Of Anverene D		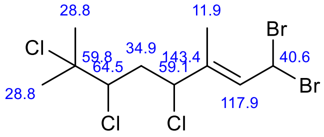
Theoretical<br>Regio-Isomer<br>Of Anverene D		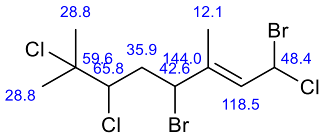
Theoretical<br>Regio-Isomer<br>Of Anverene D		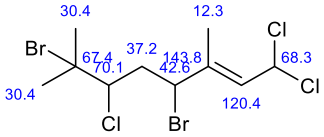
Theoretical<br>Regio-Isomer<br>Of Anverene D		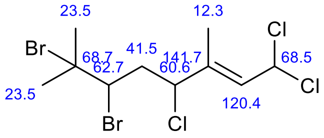
(**9**)<br>Anverene E	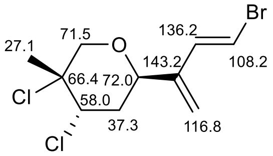	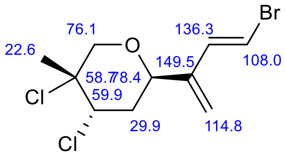
Theoretical<br>Regio-Isomer<br>Of Anverene E		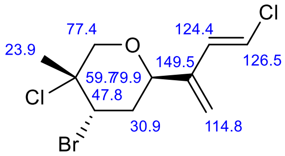
Theoretical<br>Regio-Isomer<br>Of Anverene E		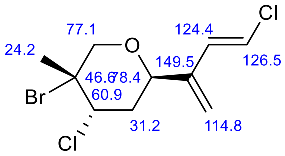
